# *Arabidopsis thaliana* 3-mercaptopyruvate sulfurtransferases interact with and are protected by reducing systems

**DOI:** 10.1016/j.jbc.2021.100429

**Published:** 2021-02-17

**Authors:** Anna Moseler, Tiphaine Dhalleine, Nicolas Rouhier, Jérémy Couturier

**Affiliations:** Université de Lorraine, INRAE, IAM, Nancy, France

**Keywords:** sulfurtransferase, hydrogen sulfide, persulfide, thioredoxin, glutathione, cysteine, 3-mercaptopyruvate, ABA, abscisic acid, BiFC, bimolecular fluorescence complementation, CARS, cysteinyl-tRNA synthetase, DES1, cysteine desulfhydrase 1, *E. coli* SseA, *Escherichia coli* 3-mercaptopyruvate sulfurtransferase, GRX, glutaredoxin, GSSH, glutathione persulfide, H_2_S, hydrogen sulfide, MST, 3-mercaptopyruvate sulfurtransferase, NaHS, sodium hydrosulfide, NTR, NADPH thioredoxin reductase, NTRB, NAPDH thioredoxin reductase B, PTM, posttranslational modification, Rhd, rhodanese, roGFP, redox-sensitive green fluorescent protein, STR, Sulfurtransferase, TRX, thioredoxin, TUM1, thiosulfate sulfurtransferase 1

## Abstract

The formation of a persulfide group (-SSH) on cysteine residues has gained attention as a reversible posttranslational modification contributing to protein regulation or protection. The widely distributed 3-mercaptopyruvate sulfurtransferases (MSTs) are implicated in the generation of persulfidated molecules and H_2_S biogenesis through transfer of a sulfane sulfur atom from a suitable donor to an acceptor. *Arabidopsis* has two MSTs, named STR1 and STR2, but they are poorly characterized. To learn more about these enzymes, we conducted a series of biochemical experiments including a variety of possible reducing systems. Our kinetic studies, which used a combination of sulfur donors and acceptors revealed that both MSTs use 3-mercaptopyruvate efficiently as a sulfur donor while thioredoxins, glutathione, and glutaredoxins all served as high-affinity sulfane sulfur acceptors. Using the redox-sensitive GFP (roGFP2) as a model acceptor protein, we showed that the persulfide-forming MSTs catalyze roGFP2 oxidation and more generally *trans*-persulfidation reactions. However, a preferential interaction with the thioredoxin system and glutathione was observed in case of competition between these sulfur acceptors. Moreover, we observed that MSTs are sensitive to overoxidation but are protected from an irreversible inactivation by their persulfide intermediate and subsequent reactivation by thioredoxins or glutathione. This work provides significant insights into *Arabidopsis* STR1 and STR2 catalytic properties and more specifically emphasizes the interaction with cellular reducing systems for the generation of H_2_S and glutathione persulfide and reactivation of an oxidatively modified form.

An ever-growing body of evidence indicates that hydrogen sulfide (H_2_S) plays a role in cellular signaling as other gaseous molecules such as nitric oxide (NO) and carbon monoxide (CO). Signaling by H_2_S is proposed to occur *via* the posttranslational modification (PTM) of critical cysteine residues (RSH) to persulfides (RSSH), called persulfidation, resulting in a cysteine whose thiol group is covalently bound to sulfur (sulfane sulfur) (1). Oxidized thiol species such as sulfenic acids (RSOH), but not reduced thiols, are the direct targets of H_2_S reactivity (2, 3, 4). Cysteine persulfides have been found in free cysteine, small molecule peptides, as well as in proteins (5). Recently, it was demonstrated that prokaryotic and mammalian cysteinyl-tRNA synthetases (CARSs) have a crucial role in the synthesis of cysteine persulfides (Cys-SSH) (6, 7) thus representing one of the numerous pathways contributing to the formation of persulfides.

In plants, H_2_S is associated with various physiological functions ranging from responses to abiotic and biotic stresses, plant development (seed germination, root development, leaf senescence), photosynthesis, and autophagy to stomatal movement (8). Three distinct enzymatic pathways producing H_2_S have been identified. Sulfide is primarily produced in chloroplasts through the action of sulfite reductase during the reductive assimilation of sulfate. It is then incorporated into the amino acid skeleton of O-acetylserine to form cysteine, the biosynthesis of which can occur in the cytosol, plastids, and mitochondria (9). Another pathway of H_2_S biogenesis is the conversion of cyanide and cysteine into β-cyanoalanine and H_2_S, which is catalyzed by the β-cyanoalanine synthase CAS-C1 in mitochondria (8, 10). In the cytosol, the L-cysteine desulfhydrase 1 (DES1) degrades cysteine into H_2_S, ammonia, and pyruvate (11, 12). Up to now, the links between the H_2_S-producing enzymes and the cellular persulfidation state have not been clearly identified in plants. Although *des1* mutant plants display a reduced sulfide production (30% decrease under steady-state growth conditions) (11) and are affected in several physiological pathways (senescence, autophagy, stomatal closure, and immunity), the number of persulfidated proteins in wildtype (WT) and *des1* plants (2015 and 2130, respectively) is similar and with a high overlap of 85% (13). In *des1* mutants, the persulfidation level of only 47 proteins, including protein kinases, protein phosphatases, and abscisic acid receptors, was decreased underlying a limited role of DES1 in protein persulfidation. Hence, other factors/pathways promoting protein persulfidation in plants remain to be identified.

In mammals, H_2_S is generated primarily by three different enzymes: cystathionine beta-synthase, cystathionine gamma-lyase, and 3-mercaptopyruvate sulfurtransferase (MST) (14, 15). MSTs belong to the sulfurtransferase (STR) family, which are characterized by the presence of a rhodanese (Rhd) domain (16, 17). Owing to the conserved catalytic cysteine present in the rhodanese domain, STRs are implicated in sulfur/persulfide trafficking through their ability to catalyze the transfer of a sulfur atom to nucleophilic acceptors. The MST isoforms are characterized by the presence of two Rhd domains with only the C-terminal one possessing the catalytic cysteine in a characteristic CG[S/T]GVT signature (17). In mammals, MSTs are found in both the cytosol and mitochondria. In rat liver, the specific MST activity is 3-fold higher in mitochondria than in the cytosol (18). In mice, the mitochondrial MST contributes to H_2_S metabolism and sulfide signaling by releasing H_2_S in the presence of a reductant such as a thioredoxin (TRX) (19, 20). Furthermore, the production of polysulfides H_2_S_2_ and H_2_S_3_ by MST has been reported in the absence of reductant (21), whereas Cys-SSH and glutathione persulfide (GSSH) were observed in the presence of physiological concentrations of cysteine and GSH (22). Similar results were observed in *Escherichia coli* with the involvement of the MST ortholog, SseA, in the production of reactive sulfane sulfur and notably GSSH and GSSSH (23). From a physiological point of view, the levels of total persulfidated species in the brain of MST-KO mice are less than 50% of those in the brain of WT mice indicating that mitochondrial MST is indeed an important factor in promoting protein persulfidation (21, 22). The biochemical characterization of human MSTs has confirmed that TRXs are physiological persulfide acceptors contributing to the generation of H_2_S and thus TRXs can be considered as regulators of protein persulfide levels in the cells (24).

Although good evidence for the function of MSTs in sulfur transfer or H_2_S synthesis has been gained in bacteria and vertebrates over the last decade, their function in plants is just being elucidated. Plants possess at least one MST isoform, but *Arabidopsis thaliana* has two MSTs, named STR1 and STR2, which are located in mitochondria and cytosol, respectively (25, 26). In *Arabidopsis*, the MSTs are suggested to be multifunctional enzymes involved in cysteine catabolism and sulfide production and possibly in cyanide detoxification as shown *in vitro* (27, 28, 29, 30). Regarding the MST role in cysteine degradation in mitochondria, STR1 converts 3-MP, formed by the action of a yet unknown cysteine aminotransferase on cysteine, to pyruvate resulting in the formation of an enzyme-bound persulfide (Fig. S1). It is suggested that STR1 persulfide is then transferred to GSH (28). In accordance with their catalytic mechanism, both *Arabidopsis* MSTs were isolated as persulfidated proteins from leaf extracts (13). In addition to these functions, an interaction of both MSTs was observed with TRXs through bimolecular fluorescence complementation (BiFC), but no further investigation of the implication of these two systems in H_2_S biosynthesis and protein persulfidation has been performed (31).

In this study, we have investigated the kinetics of H_2_S biosynthesis and of low-molecular-weight persulfide production from 3-MP catalyzed by recombinant *Arabidopsis* STR1 and STR2 since a fine characterization of their enzymatic properties was not achieved so far. This 3-MP conversion constitutes an efficient H_2_S biogenesis system in the presence of the major cellular reductants (TRX, GRX). Other *trans*-persulfidation reactions, not necessarily releasing H_2_S, have been observed with GSH, Cys, or the model acceptor protein roGFP2, suggesting that MSTs could participate in the trafficking of sulfane sulfur and/or in protein oxidation. These results advance our understanding of the roles of these two MSTs. The observed partnership with physiological sulfur acceptors such as TRXs, GRXs, and GSH will help mapping sulfur transfer events across interconnected pathways and designing adequate strategies for studying H_2_S biogenesis *in planta*.

## Results

### STR1 and STR2 favor 3-MP as sulfur donor to form persulfides

**Figure 1:**
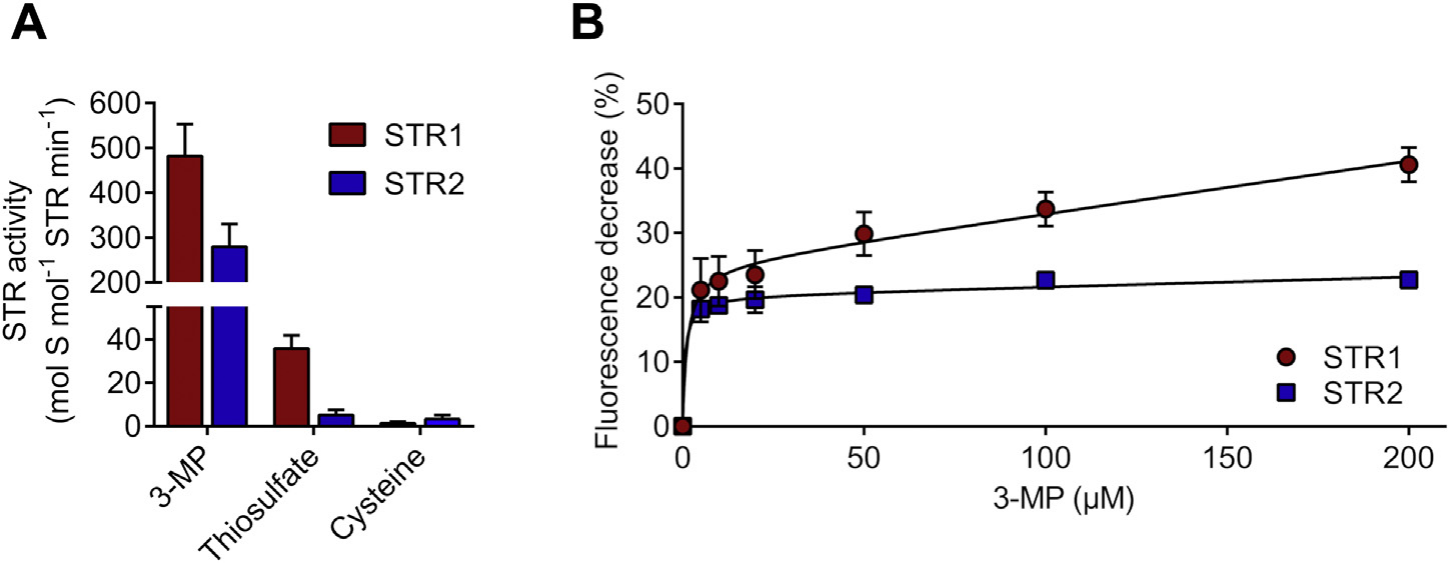
**Substrate specificity of STR1 and STR2.** The substrate specificity (*A*) of STR1 and STR2 toward 3-MP, thiosulfate, and cysteine was evaluated by determining STR activity in the presence of 250 μM sulfur donor and 1 mM DTT. Activity is expressed as mole of sulfide produced by mole enzyme per minute (*n* = 3; means ± SD). The binding affinity (*B*) of STR1 and STR2 toward 3-MP was measured in the presence of increasing concentrations of 3-MP analyzing the protein intrinsic fluorescence. The decrease of fluorescence emission at 337 nm was plotted against 3-MP concentration to determine the *K*_*d*_ value (*n* = 3; means ± SD). 3-MP, 3-mercaptopyruvate.

To analyze the functional facets of *Arabidopsis* MSTs, the corresponding recombinant proteins were purified after heterologous expression in *E. coli* with a production yield for STR1 of 20 mg and for STR2 of 4 mg of protein from 1 l bacterial culture. Different sulfur-containing compounds were tested as substrates to examine which sulfur donor is preferentially used by STR1 and STR2 *in vitro*. The protein activity was quantified by measuring the DTT-released H_2_S through the formation of methylene blue (Fig. S2) (32). The data obtained clearly indicated that 3-MP was the preferred sulfur donor that leads to H_2_S production (Fig. 1*A*). Only a very low activity was observed with thiosulfate as sulfur donor (Fig. 1*A*), although it was shown previously that STR1 possessed a thiosulfate:cyanide sulfurtransferase activity (33). No activity was measured in the presence of cysteine.

The interaction of 3-MP with both STR1 and STR2 was then evaluated using fluorescence measurements. Indeed, some non-plant MSTs exhibit quenched intrinsic fluorescence after forming the intermediate persulfide, owing to the location of a tryptophan residue close to the active site (34, 35, 36). Similar to *E. coli* SseA or human TUM1, STR1 and STR2 display intrinsic fluorescence with a maximum at 336 nm when excited at 270 nm (Fig. S3*A*). The fluorescence emission spectra of either *Arabidopsis* MSTs did not change following the addition of pyruvate but showed a strong decrease after addition of 3-MP (Fig. S3, *B*–*D*). Thus, we analyzed the fluorescence changes as a function of 3-MP concentration to determine the STR dissociation constant for 3-MP. *K*_*d*_ values of 1.3 ± 0.9 and 0.7 ± 0.3 μM were obtained for STR1 and STR2, respectively (Fig. 1*B*). In addition, STR1 C333S and STR2 C298S variants, in which the catalytically important cysteine residue present in the 6-amino acid CGTGVT signature was substituted for a serine, were generated. The intrinsic fluorescence of both variants also decreased after addition of 3-MP but not as strongly as in STR1 and STR2 indicating that the binding of 3-MP already led to fluorescence quenching, not only persulfide formation (Fig. S3, *B*–*D*).

**Figure 2:**
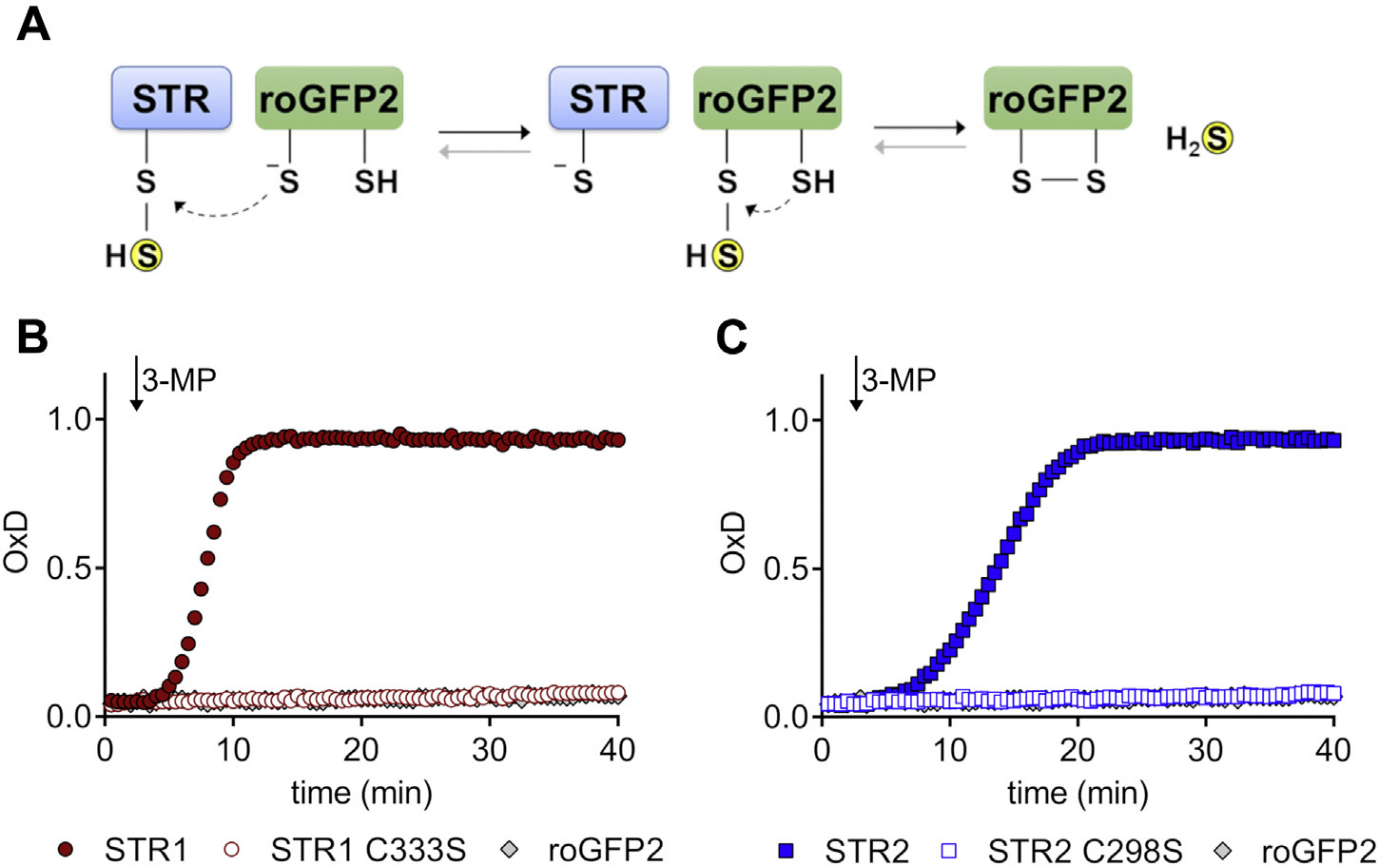
**STR1 and STR2 oxidize roGFP2 through *trans*-persulfidation reactions.** Suggested reaction mechanism of 3-mercaptopyruvate sulfurtransferase–mediated persulfidation and oxidation of roGFP2 (*A*). In the first step, the sulfur atom is transferred from 3-MP to the 3-mercaptopyruvate sulfurtransferase, resulting in a cysteine persulfide intermediate in the active site. In the second step, the sulfane sulfur might be transferred to roGFP2 and H_2_S liberated resulting in an oxidized roGFP2. The importance of the catalytic cysteine of STR1 (*B*) and STR2 (*C*) for the persulfidation and subsequent oxidation of roGFP2 was determined in the presence of 1 μM reduced roGFP2 and 50 nM STR or roGFP2 alone. The *arrow* indicates the time point of 20 μM 3-MP addition (*n* = 3). 3-MP, 3-mercaptopyruvate; STR, sulfurtransferase.

Then we examined whether MSTs are able to transfer a persulfide from 3-MP to an acceptor protein. Therefore, we used the redox-sensitive green fluorescent protein (roGFP2) as a model acceptor protein to analyze protein *trans*-persulfidation *in vitro* based on a previous work in which it was observed that roGFP2 can be oxidized through the activity of MSTs using 3-MP as sulfur donor in living cells (37). Considering that 3-MP alone does not trigger roGFP2 oxidation and that the reaction between 3-MP and MSTs leads to the persulfidation of the MST catalytic cysteine, it is likely that this persulfide group is then transferred to one of the Cys of the reduced roGFP2 and that the second Cys of roGFP2 reduces the persulfide, yielding H_2_S and an oxidized roGFP2 (Fig. 2*A*). The resulting intramolecular disulfide bridge of roGFP2 changes the steric arrangement of the beta barrel surface and in turn the optical characteristics of the chromophore enabling a direct readout of roGFP2 oxidation. Addition of 3-MP to the MST and roGFP2 mix led indeed to an efficient oxidation of roGFP2, whereas no oxidation was observed in the absence of MST or using variants mutated for the conserved active site Cys of the MST (STR1 C333S and STR2 C298S) (Fig. 2, *B* and *C*). Altogether, these findings indicate that STR1 and STR2 form persulfides on the catalytic cysteine after treatment with 3-MP, which is the canonical substrate *in vitro* and is able to transfer the persulfide to an acceptor.

### STR1 and STR2 efficiently produce H_2_S in the presence of various physiological acceptors

Earlier it was suggested that STR1 plays a role in cysteine degradation by transferring a persulfide from 3-MP to GSH (28), and in addition, it was reported through BiFC that STR1 and STR2 interact with the mitochondrial TRXo1 or cytosolic TRXh1, respectively (31). These results prompted us to investigate the interaction of the MSTs with a larger panel of physiologically relevant sulfane sulfur acceptors, GSH and cysteine, and also TRX or GRX reducing systems and to determine the steady-state kinetic parameters associated with H_2_S generation (Table 1). In all cases, a hyperbolic relationship between the rate of reaction and the concentration of acceptor was obtained (Fig. S4, *A*–*E*). Catalytic efficiencies (*k*_cat_/*K*_*m*_) ranging from 5.2 × 10^5^ to 8.7 × 10^6^ M^−1^ s^−1^ have been measured in the presence of the TRX or GRX reducing systems for both STR1 and STR2, which are 10- to 100-fold higher than the values obtained with GSH or cysteine (Table 1). The apparent *K*_m_ values for the two mitochondrial TRXo1 and TRXo2 as well as the cytosolic TRXh1 were in the low micromolar range ranging from 1.3 to 5.3 μM. Similar apparent *K*_m_ values around 3 μM were recently estimated for the human MPST1 and MPST2 with the cytosolic thioredoxin TXN (24). In contrast, the apparent *K*_m_ values of STR1 or STR2 for GSH, 200 or 350 μM respectively, differ considerably from the value of 28 mM obtained for human MPST2 (38). Of interest, apparent *K*_m_ values of 0.7 to 4.6 μM were measured for GRXs (GRXC1 and C4) using both STRs indicating that MST activity can be also coupled to these reductases. Similar steady-state kinetic parameters were determined for STR1 using TRXo1, GRXC1, or GSH by following the NADPH consumption instead of measuring the methylene blue formation (Fig. S5). Finally, the apparent *K*_m_ values for 3-MP were estimated at saturating concentrations of each acceptor using mitochondrial TRXo1 and TRXo2 for STR1 and the cytosolic TRXh1 for STR2 and ranged from ∼290 to ∼460 μM (Table 2, Fig. S4*F*). Altogether, these results show that both *Arabidopsis* MST isoforms have globally similar kinetic constants for each substrate or reducing system tested. The apparent affinities indicate that all these interactions may be physiologically relevant and point for the first time to a possible role of GRXs. Moreover, GSH may have an important role as an acceptor of persulfides from plant MSTs, unlike human MPST.

**Table 1:** Kinetic parameters of 3-MP sulfurtransferase activity of *Arabidopsis* STR1 and STR2 using distinct sulfur acceptors

Acceptor	STR1			STR2		
	*K*_*m*_ (μM)	*k*_cat_ (s^−1^)	*k*_cat_/*K*_*m*_ (M^−1^ s^−1^)	*K*_*m*_ (μM)	*k*_cat_ (s^−1^)	*k*_cat_/*K*_*m*_ (M^−1^ s^−1^)
TRXo1	5.3 ± 1.0	6.8	1.3 × 10^6^	na	na	na
TRXo2	1.3 ± 0.2	3.6	2.8 × 10^6^	na	na	na
TRXh1	na	na	na	1.4 ± 0.2	1.4	1.0 × 10^6^
GRXC4	1.1 ± 0.1	4.6	4.2 × 10^6^	4.6 ± 0.6	2.4	5.2 × 10^5^
GRXC1	0.7 ± 0.1	6.1	8.7 × 10^6^	1.43 ± 0.3	3.1	2.2 × 10^6^
GSH	200 ± 20	5.3	2.7 × 10^4^	350 ± 10	2.3	6.6 × 10^3^
Cysteine	2200 ± 400	3.9	1.7 × 10^3^	1300 ± 70	1.5	1.2 × 10^3^

na, not analyzed.

Steady-state kinetic parameters were determined by varying the acceptor concentration at a saturating concentration of 3-MP (*n* = 3; means ± SD).

**Table 2:** Kinetic parameters of sulfurtransferase activity of *Arabidopsis* STR1 and STR2 for 3-MP

Acceptor	STR1			STR2		
	*K*_*m*_ (μM)	*k*_cat_ (s^−1^)	*k*_cat_/*K*_*m*_ (M^−1^ s^−1^)	*K*_*m*_ (μM)	*k*_cat_ (s^−1^)	*k*_cat_/*K*_*m*_ (M^−1^ s^−1^)
TRXo1	286 ± 5	13.5	4.7 × 10^4^	na	na	na
TRXo2	456 ± 24	9.7	2.1 × 10^4^	na	na	na
TRXh1	na	na	na	377 ± 15	9.7	2.5 × 10^4^

na, not analyzed.

Steady-state kinetic parameters were determined by varying the 3-MP concentration at a saturating concentration of acceptors (*n* = 3; means ± SD).

**Figure 3:**
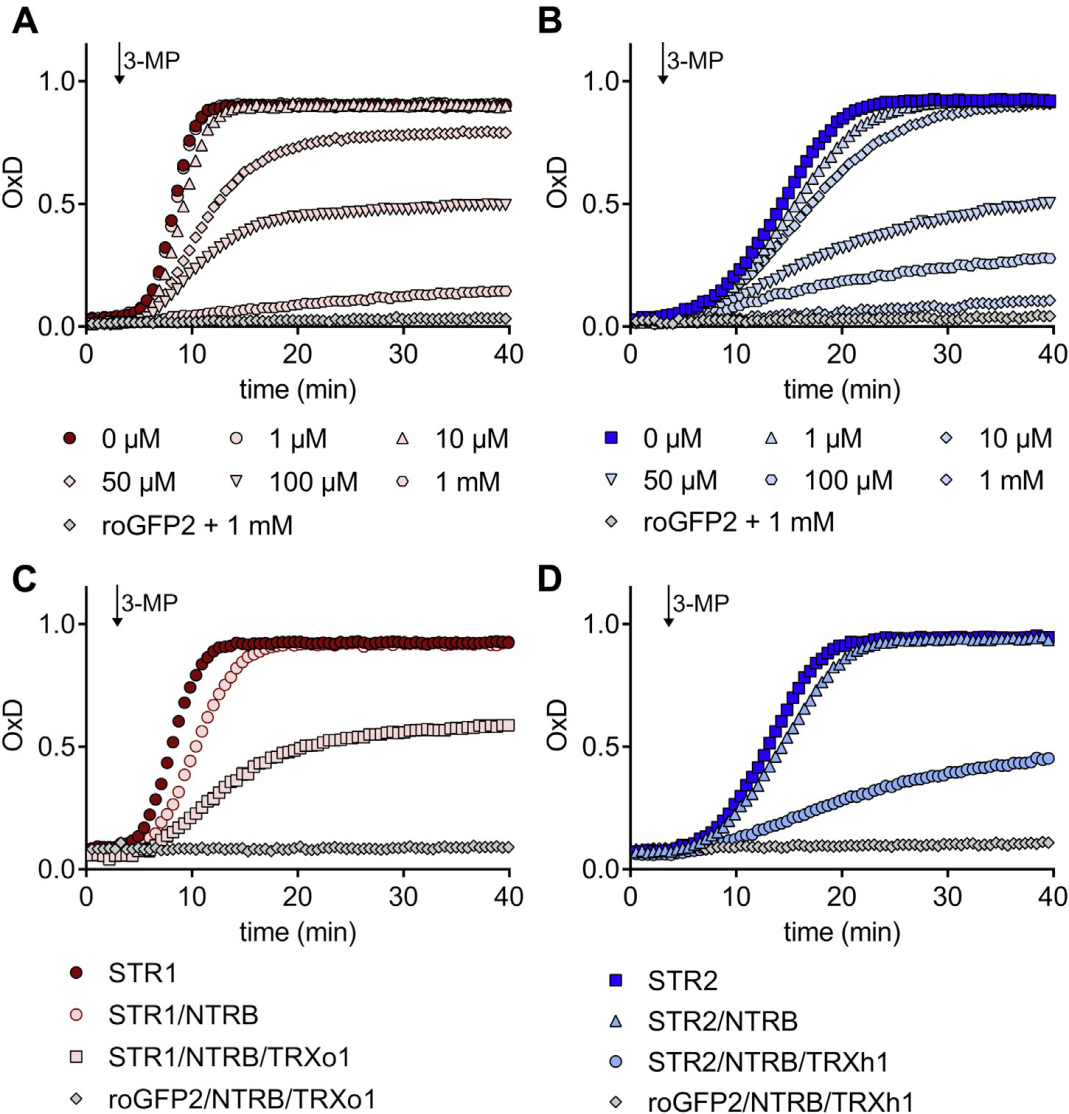
**Both TRX and GSH systems prevent the 3-mercaptopyruvate sulfurtransferase–dependent oxidation of roGFP2.** The impact of each reducing system was determined by using 1 μM reduced roGFP2 in the presence of 50 nM STR1 (*A*) or STR2 (*B*) and different amounts of GSH (0–1 mM GSH) or 200 μM NADPH, NADPH/200 nM NTRB, or NADPH/NTRB/1 μM TRXo1 for STR1 (*C*) and 200 μM NADPH, NADPH/200 nM NTRB, or NADPH/NTRB/1 μM TRXh1 for STR2 (*D*). The fluorescence of roGFP2 was followed over time, and the arrow indicates the time point of 20 μM 3-MP addition (*n* = 3). 3-MP, 3-mercaptopyruvate; NTRB, NAPDH thioredoxin reductase B; OxD, degree of oxidation.

The ability of both MSTs to oxidize roGFP2 through *trans*-persulfidation reactions prompted us to analyze whether reducing systems could prevent or compete with this sulfur transfer reaction. In the presence of 1 to 1000 μM GSH, addition of 3-MP led to a minor increase in oxidation of roGFP2 compared with the degree of oxidation in the absence of GSH indicating that GSH indeed competed with roGFP2 as persulfide acceptor (Fig. 3, *A* and *B*, Fig. S6). Similar results were obtained when the TRX system replaced GSH. After addition of 3-MP, a marginal oxidation of roGFP2 was observed in the presence of the complete TRX reducing system in contrast to controls in which just NADPH or NADPH and NAPDH thioredoxin reductase B (NTRB) were present (Fig. 3, *C* and *D*). In the presence of NTRB the oxidation of roGFP2 was nevertheless lower indicating that NTRB can reduce to some extent a persulfidated roGFP2 or STR1. Altogether, these results demonstrate that STR1 and STR2 promote preferentially the persulfidation of TRXs and GSH to generate H_2_S or GSSH rather than the persulfidation of roGFP2.

### Only the reactive catalytic cysteine of MSTs is indispensable to their STR activity

**Figure 4:**
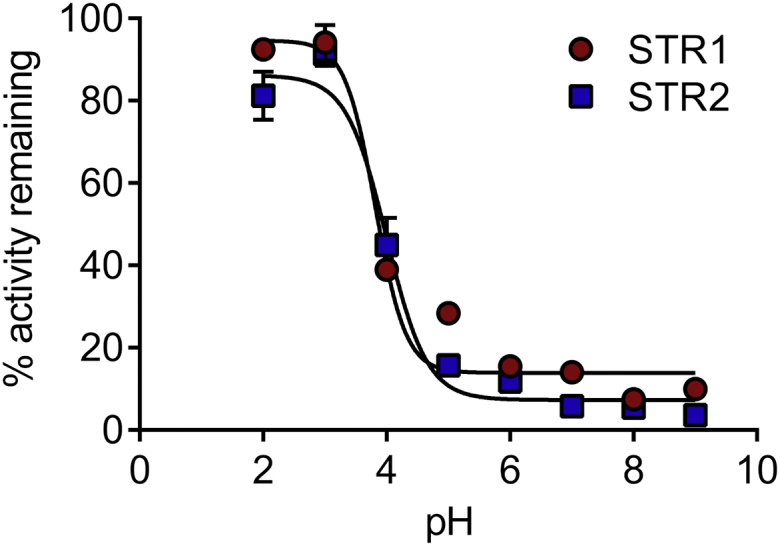
**The catalytic cysteines of STR1 and STR2 have low p*K***_**a**_**.** Reduced STRs were incubated with or without 2 mM H_2_O_2_ in different buffers ranging from pH 2.0 to 9.0 prior to measurement of their activity by using the methylene blue assay. The percentages of remaining activity at each pH were determined by comparing the activity of the enzyme incubated with and without H_2_O_2_ (n = 3; means ± SD).

STR1 and STR2 contain five and four cysteines, respectively. Similar to MSTs from other organisms, the C-terminal Rhd-domain of STR1 and STR2 contains the conserved active site cysteine in a CG[T/S]GVT motif (17). The catalytic efficiency of MSTs should be at least partially governed by the reactivity of the catalytic cysteine, which is dependent on its p*K*_*a*_. Seeking to assess the p*K*_*a*_ of Cys333 of STR1 and Cys298 of STR2, we used a method relying on H_2_O_2_ as blocking reagent reacting with thiolates but not thiols. The pH-dependent inactivation of STRs by H_2_O_2_ was followed by comparing their residual activity in the presence of 3-MP and DTT as reductant, after preincubation of the reduced proteins in different buffers ranging from pH 2.0 to 9.0 with or without H_2_O_2_. From these titration curves, we obtained a p*K*_*a*_ value for STR1 of 3.8 ± 0.1 and for STR2 of 4.0 ± 0.1 (Fig. 4). These results indicate that, at physiological pH, the thiolate form will be the dominant, almost only, species existing for the catalytic cysteine of both STRs.

**Figure 5:**
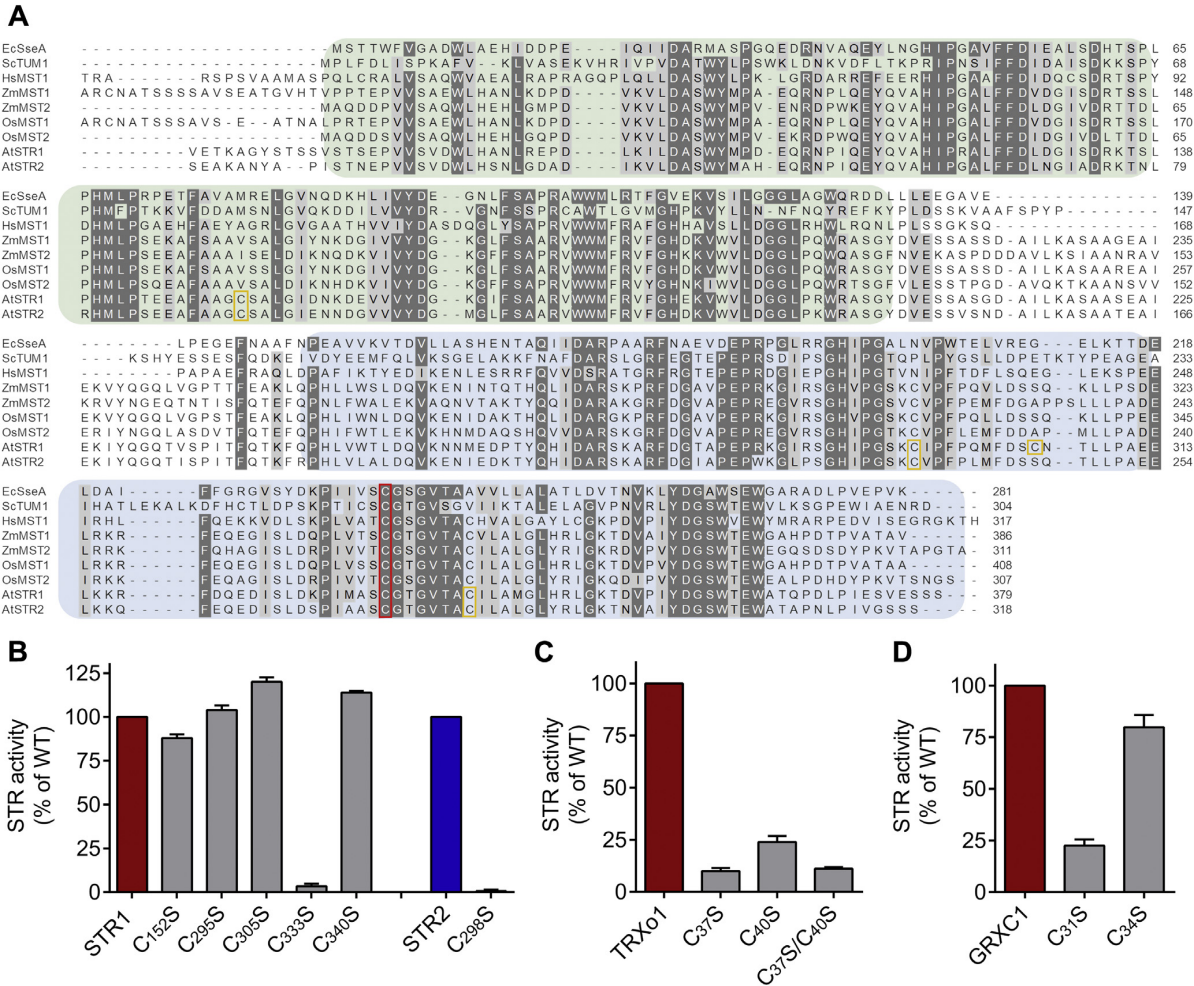
**Involvement of MST cysteines in 3-mercaptopyruvate conversion and interaction with cellular reducing systems.***A*, amino acid sequence alignment of MSTs. Shown are the two Rhd domains with the inactive domain in *green* and the active domain in *blue*. The catalytic cysteine of the active site motif is indicated by a *red square*, and other cysteines found in *Arabidopsis* MSTs are indicated by an *orange square*. The sequence alignment was performed with MUSCLE. Sequences used are *Ec*SseA (NP_417016), *Sc*TUM1 (NP_014894), *Hs*MST1 (NP_066949), *Zm*MST1 (GRMZM2G029262), *Zm*MST2 (GRMZM2G168888), *Os*MST1 (Os12g41500), *Os*MST2 (Os02g07044), *At*STR1 (AT1G79230), *At*STR2 (AT1G16460). *B*, the importance of each cysteine residue of STR1 or STR2 was evaluated by determining the STR activity of the respective monocysteinic variants in the presence of 250 μM 3-MP, 200 μM NADPH, 200 nM NTRB, and 30 μM TRXo1 (for STR1) or 10 μM TRXh1 (for STR2) (*n* = 3; means ± SD). *C*, the importance of the catalytic and resolving cysteines of TRXo1 for the activity of STR1 was determined in the presence of 250 μM 3-MP, 10 nM STR1, as well as 25 μM TRXo1 and 200 μM NADPH, 200 nM NTRB (*n*= 3; means ± SD). *D*, the importance of the catalytic and resolving cysteines of GRXC1 for the activity of STR1 was determined in the presence of 250 μM 3-MP, 10 nM STR1, as well as 25 μM GRXC1 and 250 μM NADPH, 250 μM GSH, and 0.5 U GR. GSH-dependent activity was subtracted to analyze the additional effect of GRX (*n* = 3; means ± SD). MST, 3-mercaptopyruvate sulfurtransferase; NTRB, NAPDH thioredoxin reductase B; STR, sulfurtransferase.

Other cysteines present in plant MSTs are usually not conserved in non-plant MSTs. One is present in the N-terminal domain, and the others are present in the C-terminal domain (Fig. 5*A*). To determine whether and how they contribute to the catalytic mechanism of the respective STR, single Cys-to-Ser variants were generated. For STR2, however, only a variant (C298S) for the conserved catalytic cysteine was analyzed because other variants were expressed as insoluble proteins. Using variants mutated for the conserved active site Cys in both STRs, no H_2_S was produced from 3-MP in the presence of TRXs, which underlines its essential role in the reaction mechanism (Fig. 5*B*). In contrast, STR1 variants mutated for the nonconserved C152, C295, C305, or C340 produced similar amounts of H_2_S in our assay, indicating that these cysteines are not important for 3-MP desulfuration.

To gain more insight into the mechanism of MST-dependent H_2_S biogenesis through reduction by cellular reducing systems, we measured the activity of STR1 in the presence of single Cys-to-Ser variants of TRX and GRX active site motifs and compared the amount of released H_2_S using the respective reducing system. An important reduction of H_2_S release (Fig. 5*C*) was observed using either a variant of the catalytic (TRXo1 C37S) or of the resolving cysteine (TRXo1 C40S) of TRXo1, indicating that both cysteines of TRX are required for persulfide transfer from STR1 and persulfide reduction (Fig. S7*A*). The residual activity obtained with TRXo1 C37S and C37S/C40S variants suggests that NTRB reduces to some extent a persulfidated STR1 and the slightly higher activity obtained with TRXo1 C40S that NTRB reduces to some extent a persulfidated TRXo1. Increasing the amount of NTRB in the absence of TRX, however, did not increase STR activity (Fig. S7*B*). The necessity of both Cys for full activity was also observed with the monocysteinic variants of TRXo2 supporting a dithiol mechanism for the TRX-mediated H_2_S release from STRs (Fig. S7, *A* and *C*). Regarding the TRX system, the reaction mechanism is proposed to occur *via* a ping-pong mechanism in which 3-MP binds to the MST and an enzyme-bound persulfide intermediate is formed (1, 36, 38). Following the release of pyruvate, the persulfide is transferred to the catalytic cysteine of the TRX. In the next step, the resolving cysteine of the TRX active site signature reduces the mixed disulfide, yielding H_2_S and an oxidized TRX, which is recycled *via* TRX reductase and NADPH (Fig. S7*, A*) (24). Of interest, analyses of the monocysteinic variants of GRXC1, which also possesses a CxxC active site motif as TRXs, revealed that only the catalytic cysteine (GRXC1 C31) is essential for the release of H_2_S. Indeed, mutation of the resolving cysteine (GRXC1 C34) did not influence much the amount of released H_2_S compared with the native GRXC1 (Fig. 5*D*). Similar results with the monocysteinic variants of GRXC1 were obtained using STR2. Hence, the GRX-mediated H_2_S release from STRs is based on a monothiol mechanism with GSH serving to recycle the catalytic cysteine, even though we cannot exclude a contribution of the second resolving cysteine when present (Fig. S7, *D* and *E*).

### The persulfide intermediate of MSTs protects them from H_2_O_2_-dependent inactivation

Since p*K*_a_ measurements indicated that the STR1/2 catalytic cysteine is sensitive to H_2_O_2_ inactivation, we wanted next to assess whether having a catalytic persulfide intermediate could have a protective effect. First, we analyzed the influence of the redox state of STR1 on substrate consumption. Therefore, reduced and persulfidated STR1, treated or not with H_2_O_2_, were incubated with 3-MP in the absence of reductant and product formation was detected by HPLC. The methylene blue method employed previously for activity measurements was not used owing to the requirement of a reductant, which would have interfered with the redox state of the persulfidated protein. Pyruvate was detected in the presence of both reduced and persulfidated STR1 indicating conversion of 3-MP to pyruvate (Fig. S8, *A* and *C*). In contrast, pretreatment of the reduced as well as persulfidated STR with H_2_O_2_ prevented the reaction (Fig. S8, *B* and *D*). These results demonstrated that persulfidated STR1 was still active in the absence of reductant but its activity is inhibited by H_2_O_2_ as observed for a reduced STR1.

**Figure 6:**
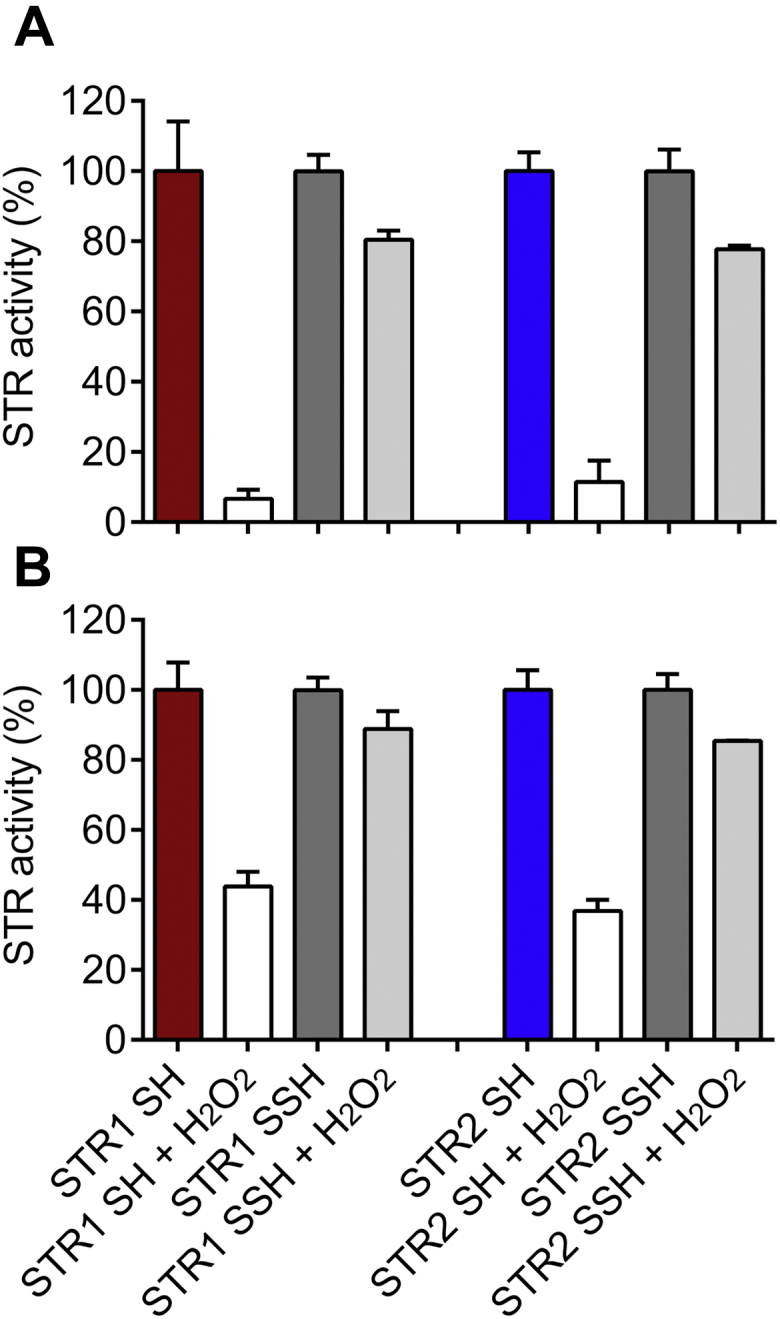
**Reduced but not persulfidated 3-mercaptopyruvate sulfurtransferases are irreversibly inactivated by H**_**2**_**O**_**2**_**.** After preincubation of 2 μM STR with 2 mM H_2_O_2_ for 3 min at 25 °C, the STR activity was measured with 20 nM STR in the presence of 250 μM 3-mercaptopyruvate and GSH/GR system (1 mM GSH, 250 μM NADPH, and 0.5 U GR) (*A*) or TRX system (10 μM TRX [TRXo1 for STR1 and TRXh1 for STR2], 200 μM NADPH, and 200 nM NTRB) (*B*). The values are expressed as percentages of the activity of reduced STRs (STR SH = 100%) (*n* = 3; means ± SD). NTRB, NAPDH thioredoxin reductase B; STR, sulfurtransferase.

Recently, Dóka and colleagues showed that a persulfidation step prevented the irreversible oxidation of the human serum albumin used as a protein model (39). Therefore, we further compared the sensitivity toward H_2_O_2_ inhibition of STR1 and STR2 using either reduced or persulfidated forms and the reactivation of their activity by reducing systems. Incubating reduced STR1 and STR2 with an excess of H_2_O_2_ for 3 min before measuring activity in the presence of the physiological GSH/GR or TRX reducing systems decreased activities up to 60% to 90% compared with untreated proteins. This loss of activity indicated that a predominantly irreversible oxidation of the catalytic cysteine occurred during the pretreatment and that none of the reductants could reduce and fully reactivate the MSTs (Fig. 6). In contrast, the activity recovery assays indicated that both persulfidated MSTs treated with H_2_O_2_ were mostly reversibly oxidized (Fig. 6). The incomplete reactivation compared with the nontreated persulfidated MSTs is likely due to the incomplete initial persulfidation of STRs. Altogether, these data show that the persulfidation of MSTs protects them against the irreversible oxidation of their catalytic cysteine by H_2_O_2_ and that both GSH and TRX can reduce not only RSSH species but also RSSO(n)H species resulting from the oxidation of persulfide groups by H_2_O_2_ (15).

## Discussion

### *Arabidopsis* MSTs share common properties with non-plant orthologs

In the *A. thaliana* genome 21 STRs are annotated, but their specific functions are yet largely unknown (40). The mitochondrial STR1 and cytosolic STR2 are the sole MSTs (17). Accordingly, the *in vitro* activity of STR1 and STR2 was much higher when 3-MP was used as sulfur donor in comparison with thiosulfate (Fig. 1), which is in line with previous results obtained on STR1 (31, 37, 41) and *E. coli* SseA (34) but different from mouse MST for which H_2_S-producing activities were the same using 3-MP and thiosulfate (20). Both STR1 and STR2 displayed *K*_*d*_ values for 3-MP close to 1 μM (1.3 ± 0.9 and 0.7 ± 0.3 μM, respectively, Fig. 1*B*), which are in the same range as the *K*_*d*_ value of 5 ± 0.2 μM determined for *E. coli* SseA (34). Despite the presence of several cysteine residues in their primary sequence, only the catalytic cysteine is indispensable for the catalytic activity of STR1 and STR2 (Fig. 4). Accordingly, p*K*_a_ values of 3.8 ± 0.1 and 4.0 ± 0.1, respectively, have been measured for the catalytic cysteine of both STR1 and STR2, values close to the one measured for human MST (5.2 ± 0.1) (36). Also, consistent with the known catalytic mechanism of MSTs, STR1 and STR2 are persulfidated in the presence of 3-MP and were identified in an *Arabidopsis* persulfidome (13). Using roGFP2 as a model acceptor protein, we confirmed that both STR1 and STR2 were able to catalyze *trans*-persulfidation (*i.e.*, sulfur transfer from a donor to an acceptor) reaction from 3-MP to a protein acceptor that is not a TRX (Fig. 2). In a similar manner, the yeast MST isoform, TUM1, acts as a sulfur carrier between the cysteine desulfurase Nfs1 and Uba4, another Rhd-containing protein implicated in tRNA thio-modification (42). This sulfur relay system is conserved in human where MST1 interacts with NFS1 and CNX5 (the human ortholog of Uba4) suggesting a dual function of MST1 both in sulfur transfer for the biosynthesis of molybdenum cofactor (Moco) and tRNA thio-modification in cytosol (35). Up to now, no physiological evidence has confirmed the existence of a similar sulfur relay in plants. Moreover, a role of STR2 in Moco biosynthesis and/or tRNA thiolation appears unlikely since *str2* mutant lines have no phenotype, whereas *cnx5* mutants are strongly affected, exhibiting a dwarf phenotype with slightly green and morphologically aberrant leaves (29, 43). From a more general point of view, despite the existence of long lists of possible persulfidated proteins in plants (13) as in other organisms (4, 44), it is yet unknown to which extent MSTs serve as a sulfur relay/trafficking protein participating in protein persulfidation.

### *Arabidopsis* MSTs efficiently produce H_2_S through their interaction with TRXs and GRXs

The interaction of mammalian or bacterial MSTs with TRXs was reported to serve for the biogenesis of H_2_S (20, 24, 36, 38). In addition, STR1 was reported to interact with the mitochondrial TRXo1 and STR2 with the cytosolic TRXh1 using BiFC studies performed in *Arabidopsis* protoplasts (31). Our *in vitro* activity assays confirmed these interactions and additionally showed that STR1 also interacts with the mitochondrial TRXo2 (Table 1). All plant MST-TRX couples studied in this work (*i.e.*, STR1-TRXo1, STR1-TRXo2, and STR2-TRXh1) catalyzed the formation of H_2_S from 3-MP (Table 1, Fig. S4). Kinetic parameters of these reactions including the apparent *K*_*m*_ values for TRX and 3-MP are in the same range as those determined for human MST1 and MST2 orthologs (24, 38). Nevertheless, the catalytic efficiency of both STR1 and STR2 (1 × 10^6^–2.8 × 10^6^) is 10- to 20-fold higher than human MSTs (1.3–1.4 × 10^5^) suggesting that plant MSTs are more efficient and/or that TRXs are more efficient physiological sulfur acceptors (Table 1). A preferential interaction of STR1 and STR2 with TRXs was also evident from the inhibition effect of TRXs in the MST-mediated roGFP2 oxidation assay (Fig. 3). Canonical TRXs are characterized by the presence of two cysteine residues in their active site, both of them being indispensable for their activity. Accordingly, a decreased H_2_S release was observed in the presence of TRX variants mutated for either cysteines (Fig. 5). Hence, the reaction of STR1 with dithiol TRXs results in the formation of a persulfide intermediate on the TRX catalytic Cys, which rapidly evolves to generate a disulfide-bridged TRX and to release H_2_S. Of interest, the catalytic efficiency of the reactions in the presence of GRXs is similar to those achieved in the presence of TRXs (Table 1). These results illustrate that GRXs also represent potential partners of MSTs for H_2_S biosynthesis. Unlike TRXs, only the catalytic cysteine is required for the GRX activity at least in the presence of GSH (Fig. S7*D*) (45, 46). Hence, once the catalytic cysteine of GRX is persulfidated, several possibilities exist considering monothiol or dithiol GRXs (Fig. S7*E*). For dithiol GRXs, the persulfide reduction by the second active cysteine might release H_2_S directly without the intervention of GSH. The reduction of the persulfidated GRXs by GSH would lead to the formation of GSSH and possibly to H_2_S release upon reaction with a second GSH molecule. The latter pathway applies for monothiol GRXs. From a physiological point of view, no MST-GRX interaction was described so far since studies concentrated mostly on the mitochondrial STR1 and there is no class I GRX in *Arabidopsis* mitochondria (47, 48, 49). However, such an interaction would be relevant for the cytosolic STR2.

In any case, the interaction of the STRs with both reducing systems occurs *via* protein persulfidation and may lead to the production of the signaling molecule H_2_S. Given the stable but reversible nature of protein persulfides, a cellular depersulfidation mechanism *via* a depersulfidase should prevent the accumulation of persulfides. For instance, TRX efficiently reduces the persulfidated cysteine not only in protein-tyrosine phosphatase 1B (PTP1B) and human and bovine serum albumin (HSA and BSA) but also in free Cys-SSH (3, 50, 51). It is noteworthy that the mammalian selenocysteine (Sec)-thioredoxin reductase exhibits itself polysulfide reducing activity *in vitro* and this is improved by the presence of TRXs (51). In contrast, increased amounts of *Arabidopsis* NTRB, which does not possess a selenocysteine residue, had no stimulatory effect on STR activity (Fig. S7*B*). Hence, in the plant system, the interaction of STR1 with TRXs rather than NTR is important. In a similar manner, the mammalian GRX/GSH system efficiently reduces both polysulfides and persulfidated BSA *in vitro* (51). The depletion or deletion of genes encoding thioredoxin reductase or glutathione reductase in animal cells is associated with increased intracellular persulfide levels (3, 39, 51). This underlines the importance of these reducing systems in protein persulfidation mechanisms, notably as a reducing mechanism that can regenerate native forms of Cys residues from persulfide species (51). Whether they act directly in the source by interfering only with persulfide-generating enzymes such as MSTs and/or whether they interact specifically with some persulfidated proteins is an important question. Of interest, in *Spinacia oleracea* seedlings treated with 100 μM NaHS, gene expression of the TRX reducing system is increased suggesting a role in H_2_S producing conditions (52). While TRXs, GSH, and GRXs are clearly important regulators of H_2_S production from persulfidated proteins including MSTs, it seems important now to analyze *in vivo* if deletion of TRXs and/or GRXs leads to increased persulfide levels in plants.

### *Arabidopsis* MSTs generate low-molecular-weight persulfides

Although the catalytic efficiency of the reaction is 100- to 1000-fold lower than values obtained with TRXs or GRXs, the ability of MSTs to release H_2_S in the presence of GSH and Cys suggests that low-molecular-weight (LMW) persulfides, such as Cys-SSH or GSSH persulfides, are formed. These data are consistent with the proposed role of STR1 in cysteine catabolism, which is to produce GSSH as a substrate for ethylmalonic encephalopathy protein 1 (ETHE1) (28). Of interest, the affinity of both *Arabidopsis* MSTs for GSH (*i.e.*, 200 and 350 μM, respectively) is much better than the one of human MST (*K_m,GSH_* of 28 mM) and thus the catalytic efficiency (*k*_cat_/*K_m,GSH_*) of *Arabidopsis* MSTs is 5000- to 20,000-fold higher (Table 1) (23, 24). Considering that GSH concentrations in the cytosol and mitochondria of plant cells are in the millimolar range (53), the reaction leading to GSSH formation should not be hampered by changes in GSH concentration or oxidation, even those occurring during oxidative stress. This should be different in mammals where the MST-dependent GSSH formation should be negligible. Also, no reaction of mouse MST with GSH was observed (20), while GSSH concentrations of 35 pmol/mg protein (7 μM) in mouse liver tissue were recently estimated by mass spectrometry (39). This GSSH production is assumed to originate from the enzymatic reaction catalyzed by the mitochondrial sulfide-quinone oxidoreductase during H_2_S oxidation (54, 55). The different properties of *Arabidopsis* MSTs may be related to the absence of sulfide-quinone oxidoreductase in plants, whereas it is present in most organisms (56).

Cysteine has been recently proposed to represent a significant sulfur acceptor of human MSTs, leading to the generation of LMW persulfides, despite poor *K*_*m*_ values (3.8–4.5 mM) and low reaction rates (1 × 10^2^ M^−1^ s^−1^) (24). Of interest, STR1 and STR2 display more favorable kinetic parameters, with *K*_*m*_ values of 1.3 to 2.2 mM and a 10-fold higher value of catalytic efficiency (Table 1). This suggests that plant MSTs may represent physiological producers of Cys-SSH in addition to GSSH. The physiological relevance of the LMW persulfide Cys-SSH, at least for mitochondria, was shown by markedly altered mitochondrial morphology in *CARS2* KO human cells or human patients with mutated *CARS*2 (6, 57). The decreased persulfide production affected also mitochondrial biogenesis and bioenergetics. Hence, Cys-SSH is metabolized through the action of the electron transport chain of mitochondria leading to the formation of H_2_S.

### Reducing systems are also involved in the redox control of *Arabidopsis* MSTs

Several PTMs of Cys residues such as glutathionylation, disulfide formation, and nitrosylation can affect the protein structure and function and play important roles in various biological contexts such as stress conditions or germination (58, 59). Similarly, protein persulfidation reversibly alters enzyme function (60). Under standard growth conditions, at least 5% of the proteome of *Arabidopsis* leaves undergo persulfidation indicating an important role of this PTM (13). The persulfidation of DES1 and NADPH oxidase RBOHD has been recently related to an increase of both protein activity in relationship with the control of ABA signaling in *Arabidopsis* guard cells (61). The NaHS-induced persulfidation promotes an increase of the catalytic activity of *Arabidopsis* glyceraldehyde-3-phosphate dehydrogenase (GAPDH) isoform C1 (GAPC1) and ascorbate peroxidase 1 (APX1) *in vitro* (62). In a similar manner, after NaHS-induced persulfidation, the catalytic activity of an animal GAPDH increases *in vitro* (63). This is consistent with the decrease of GAPDH activity in the liver of a mouse *CSE*^−/−^ null mutant, which contains markedly reduced H_2_S levels (63, 64). In contrast, other proteins like papain, a papaya proteinase I, or human tyrosine phosphatase PTP1B are inactivated through persulfide formation of an active site Cys (2, 50). For STR1 and STR2, we did not find an altered activity of persulfidated STR compared with reduced STR (Fig. 6) indicating no regulatory function of the persulfide itself on the MSTs. Already persulfidated STR1 was still able to convert 3-MP into pyruvate (Fig. S8) leading to the formation of a polysulfide. However, after H_2_O_2_ treatment, the persulfidated STR1 did not catalyze anymore 3-MP conversion into pyruvate. Presence of the GSH/GR or TRX reducing system regenerated a reduced thiol and subsequently the activity (Fig. 6). Thus, in addition to being a catalytic intermediate, the persulfide of MST may exert a protective role in oxidizing conditions. In addition, formation of a polysulfide on MSTs could also serve as a storage of sulfane sulfur. The mitochondrial STR1 was identified as a protein possessing redox-sensitive thiol residue(s) when isolated mitochondria are fed with citrate (59). The identified peptide did not include the active site Cys but C295, which is conserved in land plant orthologs, and C305, which is just present in STR1, indicating that one or both residues exhibit reactivity (17). Although a mutation of these Cys residues did not affect the 3-MP desulfuration activity of STR1 *in vitro* (Fig. 5), a regulatory role *via* the formation of a redox PTM might thus be considered.

## Conclusions

**Figure 7:**
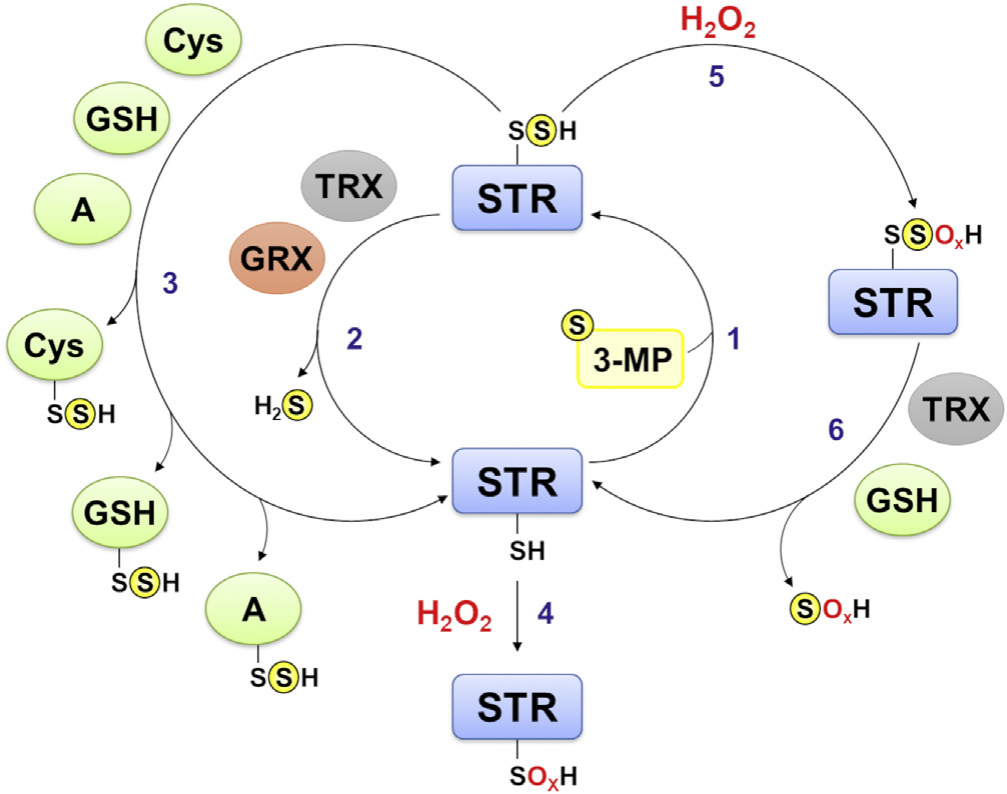
**Redox control of the activity of MSTs in *Arabidopsis***. MSTs (STR1 and STR2) preferentially use 3-mercaptopyruvate as sulfur donor (1). They are implicated in H_2_S biogenesis by interacting with TRX or GRX systems (2). By interacting with GSH and cysteine (3), both MSTs might be important for the formation of glutathione persulfide (GSSH) and cysteine persulfide (Cys-SSH). Finally, the oxidation of both reduced (4) and persulfidated catalytic cysteine (5) by H_2_O_2_ inhibits protein activity but the latter is reversed by the TRX and GSH/GR reducing systems (6). GRX, glutaredoxin; MST, 3-mercaptopyruvate sulfurtransferase; STR, sulfurtransferase; TRX, thioredoxin.

The present biochemical study of *Arabidopsis* STR1 and STR2 indicates that both MSTs use 3-MP as canonical substrate and are able to transfer the persulfide to various acceptors. The formation of the LMW persulfide, GSSH, in the presence of GSH supports a role of STR1 in cysteine degradation as previously reported (28). Furthermore, this work expands the picture, showing that both MSTs can also generate Cys-SSH and can interact with cellular reducing systems for the generation of H_2_S (Fig. 7). The interaction between MSTs and TRXs or GRXs suggests that both reducing systems play a role in the regulation of persulfide levels. Nevertheless, further work is necessary to understand how exactly persulfide levels are regulated *in vivo*. The biochemical characterization of the two *Arabidopsis* MSTs regarding their ability to transfer a persulfide together with the use of roGFP2 as a new tool to analyze MST activity provides scope for future research. Functional characterization of both MSTs has previously demonstrated that the mitochondrial STR1 contributes the main MST activity (29). Phenotypic analyses revealed that *str1* null mutants show a shrunken seed phenotype with ∼87.5% of the embryos arresting at the heart stage, whereas the residual 12.5% can develop further and show a normal development of vegetative tissue. In contrast, *str2* null mutants have no identified phenotype (29). However, the double *str1str2* mutant is not viable indicating an essential function of both STRs, which could be a direct contribution to protein persulfidation or the formation of H_2_S and/or LMW persulfides.

## Experimental procedures

### Materials

3-MP (sodium salt) was purchased from Santa Cruz Biotechnology; L-cysteine, thiosulfate, GSH, and dithiothreitol were from Sigma-Aldrich.

### Cloning and site-directed mutagenesis

The sequences coding for the presumed mature forms (*i.e.* devoid of mitochondrial targeting sequences) of *A. thaliana* STR1 (At1g79230.1) and STR2 (At1g16460.2) were cloned into the *Nco*I and *Xho*I restriction sites of pET28a and into the *Nde*I and *Bam*HI restriction sites of pET12a, respectively, to obtain untagged versions of STR1 and STR2. The *Arabidopsis* STR1 was also cloned into the pET28a vector using the STR1 rev2 primer to obtain a C-terminal His-tagged STR1 version. All cysteine residues were individually substituted into serine residues from pET28a-STR1-His or pET12a-STR2 using mutagenic oligonucleotides and the QuikChange site-directed mutagenesis kit (Agilent Technologies). The corresponding variants were named STR1 C152S, C295S, C305S, C333S, C340S and STR2 C117S, C260S, C298S, and C305S. All primers used in this study are listed in Table S1.

### Heterologous expression in *E. coli* and purification of recombinant proteins

For protein production, the *E. coli* BL21 (DE3) strain was transformed with the recombinant pET12a or pET28a plasmids, the strain expressing also the pSBET helper plasmid (encoding the AGG and AGA codon-recognizing tRNA) in combination with the pET12a plasmid. Cells were grown at 37 °C to an A_600_ of ∼0.8 in selective LB medium, and a high level of protein expression was achieved by addition of isopropyl β-D-thiogalactopyranoside (IPTG) to a final concentration of 100 μM. The cultures were harvested either after 4 h growth at 37 °C for expressing STR1 or after 16 h growth at 20 °C for expressing STR2. After centrifugation (20 min at 6380*g*), the cell pellets were resuspended in about 20 ml of TE NaCl (30 mM Tris-HCl pH 8.0, 1 mM EDTA, 200 mM NaCl) for untagged proteins or in 50 mM Tris-HCl pH 8.0, 300 mM NaCl, 10 mM imidazole for His-tagged proteins and conserved at −20 °C. Cell lysis was performed by sonication (3 × 1 min with intervals of 1 min), and the soluble and insoluble fractions were separated by centrifugation for 30 min at 27,216*g* at 4 °C.

For His-tagged proteins, the soluble fraction was loaded on a Ni^2+^ affinity column (Sigma-Aldrich). After extensive washing, proteins were eluted by adding 50 mM Tris-HCl pH 8.0, 300 mM NaCl, 250 mM imidazole. The recombinant proteins were concentrated and dialyzed by ultrafiltration under nitrogen pressure (Amicon, YM10 membrane) and stored in a TE buffer supplemented with 50% glycerol at −20 °C.

For untagged proteins, the soluble fraction was first precipitated by ammonium sulfate from 0% to 40% and then to 80% of the saturation after a centrifugation step (30 min at 27,216*g* at 4 °C). STR2 and STR1 precipitated mostly between 0% and 40% and between 40% and 80% ammonium sulfate precipitation, respectively. The precipitated fractions were subject to gel filtration chromatography (ACA34) equilibrated with TE NaCl buffer. After dialysis against TE buffer and concentration, the interesting fractions were loaded on a diethylaminoethyl sepharose column equilibrated in TE buffer. All proteins were retained and eluted using a linear 0- to 0.4 M NaCl gradient. The purest fractions as judged by SDS-PAGE gel analysis were pooled and dialyzed against TE buffer by ultrafiltration in Amicon cells equipped with a YM10 membrane. Finally, the fractions were concentrated and stored at −20 °C in the presence of 50% glycerol in addition to the buffer until further use. Protein concentrations were determined spectrophotometrically in TE buffer using molecular extinction coefficients at 280 nm of 61,670 M^−1^ cm^−1^ for STR1 and its monocysteinic variants and of 64,650 and 64,525 M^−1^ cm^−1^ for STR2 and its monocysteinic variants, respectively.

Other recombinant proteins used in this work, *e.g.*, GRXC1, GRXC4, TRXh1, and TRXh3 from poplar; NTRB, TRXo1, and TRXo2 from *A. thaliana*; as well as roGFP2, have been purified as described (45, 65, 66, 67). Glutathione reductase from baker's yeast was purchased from Sigma-Aldrich.

### Determination of kinetic constants

The STR activity was determined at 25 °C in a final volume of 400 μl of 30 mM Tris-HCl pH 8.0 by quantifying the H_2_S formed and released by a reductant using the methylene blue method. The reaction mixture contained 250 μM 3-MP and different reductants (DTT, cysteine, GSH system, GSH/GRX, and TRX systems). The GSH system was composed of 200 μM NADPH, 0.5 U GR, and 0 to 2 mM GSH, whereas the GSH/GRX system contained 200 μM NADPH, 0.5 U GR, 250 μM GSH, and 0 to 30 μM GRXC4 or GRXC1. The TRX system was composed of 200 μM NADPH, 200 nM NTRB, and 0 to 30 μM TRX. Each reducing system was incubated at 25 °C for 10 min prior to adding STR protein and 3-MP. Enzyme and substrate concentrations used are indicated in the legend of figures and tables. The reactions were stopped after 10 min by adding 50 μl of 20 mM N,N-dimethyl-p-phenylenediamine dihydrochloride (in 7.2 M HCl). The addition of 50 μl of 30 mM FeCl_3_ (in 1.2 M HCl), followed by a 20-min incubation led to formation of methylene blue, which was then measured at 670 nm. Li_2_S (1–100 μM) was used for standard curve calibration to calculate the sulfide (S) released. Activities are expressed as mol S mol^−1^ STR min^−1^. The apparent *K*_*m*_ values for sulfane sulfur acceptors were determined in the presence of 250 μM 3-MP and the following reductant concentrations: Cys (0–5 mM), GSH (0–2 mM), GRX and TRX (0–30 μM). The apparent *K*_*m*_ value for 3-MP was analyzed in the presence of 0 to 2 mM 3-MP as well as 30 μM TRXo1, TRXo2 (for STR1) or 10 μM TRXh1 (for STR2), 200 μM NADPH, and 200 nM NTRB.

For the NADPH consumption assay, 40 nM STR1 was used in the presence of 500 μM 3-MP in a final volume of 400 μl of 30 mM Tris-HCl pH 8.0. The GSH system was composed of 250 μM NADPH, 0.5 U GR, and 0 to 2 mM GSH, whereas the GSH/GRX system contained 250 μM NADPH, 0.5 U GR, 250 μM GSH, and 0 to 30 μM GRXC1. The TRX system was composed of 250 μM NADPH, 200 nM NTRB, and 0 to 30 μM TRXo1. Oxidation of NADPH was monitored at 340 nm.

Three independent reactions were performed at each reductant concentration, and apparent *k*_cat_ and *K*_*m*_ values were calculated by nonlinear regression using the Michaelis–Menten equation.

### Fluorescence properties of STRs

The intrinsic fluorescence of STR1 and STR2 and their catalytic variants, alone or with a 100-fold excess of 3-MP or pyruvate, was recorded with a Cary Eclipse spectrofluorometer (Varian) with 2 μM samples in 400 μl of 30 mM Tris-HCl pH 8.0 buffer and an excitation wavelength at 270 nm. Control spectra were run with buffer only for each sample and subtracted from the spectra.

The dissociation constants (*K*_*d*_) of STRs for 3-MP were determined in the presence of 2 μM reduced STRs and 0 to 200 μM 3-MP at 25 °C in a final volume of 30 mM Tris-HCl pH 8.0. The intrinsic fluorescence of the STR protein was immediately measured as described above. Values of fluorescence emission at 337 nm were transformed into percentage of fluorescence decrease by comparison with the value obtained for the reduced protein alone and plotted against the following nonlinear regression: Fluorescence decrease = Bmax∗[3-MP]/(*K*_*d*_+[3-MP]) + NS∗[3-MP] + Background. Here, Bmax represents the maximum specific binding, NS is the slope of the nonspecific binding line, and Background is the fluorescence from the free ligand.

### Persulfidation of STRs

Around 5 mg of STRs was reduced using 30 mM DTT in 500 μl of 30 mM Tris-HCl pH 8.0 for 1 h at 25 °C. The reduced proteins were then desalted on a G25 column pre-equilibrated with 30 mM Tris-HCl pH 8.0 buffer. Persulfidated STR1 and STR2 were prepared by incubating a reduced protein with a 10-fold excess 3-MP, respectively, for 30 min at 25 °C before desalting on G25 column.

### Redox state dependence of STR1 activity (HPLC analysis)

The influence of redox state of STR1 on its capacity to transform 3-MP into pyruvate was analyzed by incubating 500 nM STR1 (reduced, reduced treated with 5 mM H_2_O_2_, persulfidated, or persulfidated and treated with 5 mM H_2_O_2_) with 5 mM 3-MP in a final volume of 50 μl of 30 mM Tris-HCl pH 8.0 for 15 min at 25 °C. The reactions were stopped by adding 40% ethanol for 10 min before vigorous shaking for 30 s. After centrifugation (10 min at 14,000 rpm), 20 μl of supernatant was injected onto a Synergi Hydro-RP column (150 × 3 mm internal diameter, 4 μm particle size, Phenomenex) equilibrated with 20 mM phosphate buffer pH 4.6 and connected to a Shimadzu Prominence HPLC system. An isocratic elution was used at a flow rate of 0.5 ml min^−1^ and product detection was monitored at 197 nm. 3-MP and pyruvate standards were separated in similar conditions.

### Redox reactivation of oxidized STRs

Prereduced and persulfidated STRs (2 μM) were incubated with 2 mM H_2_O_2_ in 30 mM Tris-HCl pH 8.0 buffer for 3 min at 25 °C. Following this preincubation step, STR activity was determined by adding 4 μl of the preincubation mixture to the 3-MP assay described above with either 1 mM DTT, or GSH system (1 mM GSH, 200 μM NADPH, and 0.5 units GR) or TRX system (10 μM TRXo1 for STR1 and TRXh1 for STR2, 200 μM NADPH, and 200 nM NTRB) as reductants.

### p*K*_a_ determination of catalytic cysteine

The acid dissociation constant (p*K*_a_) of the catalytic cysteine of both STR1 and STR2 was determined by measuring the residual activity of reduced forms treated with H_2_O_2_ at different pH. Reduced STR, 2 μM, was incubated with or without 2 mM H_2_O_2_ in 100 mM sodium citrate, MES, phosphate or borate buffers ranging from pH 2.0 to 9.0 for 3 min. Following this preincubation step, STR activity was determined by adding 4 μl of the preincubation mixture to the 3-MP assay described above with DTT as acceptor. The percentages of remaining activity at each pH were determined by comparing the activity of the enzyme incubated with and without H_2_O_2_ and fitted to the following nonlinear regression % activity remaining = Bottom + (Top-Bottom)/(1 + 10ˆ(Log p*K*_a_ − pH × HillSlope)).

### roGFP2 interaction assay

Interaction of STRs with roGFP2 was analyzed *in vitro* by ratiometric time-course measurements on a fluorescence plate reader (EnSight multimode plate reader, PerkinElmer) with excitation at 400 ± 10 and 480 ± 10 nm and detection of emitted light at 520 nm with a bandwidth of 10 nm. Ratiometric time-course measurements were carried out with 1 μM reduced roGFP2 and 50 nM STR. For reduction of the protein, roGFP2 was incubated with 10 mM DTT for at least 20 min. The remaining DTT was removed by desalting spin columns according to the manufacturer’s manual (Zeba Spin Desalting Columns, Thermo Scientific). For interaction analysis with the TRX reducing system, 1 μM TRX, 200 nM NTRB, and 200 μM NADPH were added to the reaction mix. The reaction was started by adding 20 μM 3-MP. H_2_O_2_ and DTT were used at a final concentration of 10 mM to preset roGFP2 to the fully oxidized and fully reduced state, respectively, and to determine maximum and minimum fluorescence ratios of roGFP2 as reference values. A basal background fluorescence of buffer was subtracted from fluorescence reads for all samples. The degree of oxidation (OxD) was determined as described in Aller *et al.*, 2013 (68), according to the equation:

$${\text{OxD}}_{\text{roGFP}2}=\frac{\text{R}-{\text{R}}_{\text{red}}}{\left(\frac{{\text{I}}_{480\text{ox}}}{{\text{I}}_{480\text{red}}}\right)\left({\text{R}}_{\text{ox}}-\text{R}\right)+\left(\text{R}-{\text{R}}_{\text{red}}\right)}$$

Here, R denotes the ratio of the fluorescence intensities measured at 400 and 480 nm. R_red_ and R_ox_ represent the fluorescence ratios of fully reduced and fully oxidized roGFP2, respectively. The raw values of I were always corrected by subtracting the respective blank values.

## Data availability

All data are presented in the manuscript.

## Supporting information

This article contains supporting information (32).

## Conflict of interest

The authors declare that they have no conflicts of interest with the content of this article.
