# Supplementary material for: Arabidopsis thaliana 3-mercaptopyruvate sulfurtransferases interact with and are protected by reducing systems
Source: J Biol Chem. 2021 Feb 17;296:100429. doi: 10.1016/j.jbc.2021.100429 (PMC7995614; doi:10.1016/j.jbc.2021.100429)
Supplement: Supplemental Figures S1–S8 and Table S1 [file mmc1.pdf]

Arabidopsis thaliana 3-mercaptopyruvate sulfurtransferases interact with and are protected by reducing systems

Anna Moseler, Tiphaine Dhalleine, Nicolas Rouhier, Jérémy Couturier

SUPPORTING INFORMATION

Table S1. Primers used for cloning and site-directed mutagenesis experiments.

Figure S1. Role of 3-mercaptopyruvate sulfurtransferase (MST) in cysteine degradation.

Figure S2. Schematic representation of hydrogen sulfide quantification using the methylene blue assay.

Figure S3. Binding properties of 3-MP to STR1 and STR2.

Figure S4. 3-MP sulfurtransferase activity of STR1 and STR2 in the presence of physiological sulfane sulfur acceptors.

Figure S5. 3-MP sulfurtransferase activity of STR1 in the presence of physiological sulfane sulfur acceptors by following NADPH consumption.

Figure S6. Competition for the STR generated persulfide of different acceptors.

Figure S7. Reduction mechanism of persulfidated MSTs by TRX/GRX systems.

Figure S8. Reactivity of persulfidated STR1.

Table S1. Primers used for cloning and site-directed mutagenesis experiments.

The *Nco*I, *Nde*I, *Bam*HI and *Xho*I restriction sites used for cloning are underlined in the primers. The mutagenic codons are in bold.

Name	Sequence
STR1 for	5' CCCCCCATGGCTTCTACTGGAGTTGAG 3'
STR1 rev	5' CCCCTCGAGTTATGAAGAAGATTCAACACT 3'
STR1 rev2	5' CCCCTCGAGTGAAGAAGATTCAACACTCTC 3'
STR1 C152S for	5' GCTTTTGCTGCTGGT**TCT**TCTGCTCTTGGAATT 3'
STR1 C152S rev	5' AATTCCAAGAGCAGA**AGA**ACCAGCAGCAAAAGC 3'
STR1 C295S for	5' GTCATATACCTGGAAGCAAA**TCT**ATCCCTTTTCCTCAGATG 3'
STR1 C295S rev	5' CATCTGAGGAAAAGGGAT**AGA**TTTGCTTCCAGGTATATGAC 3'
STR1 C305S for	5' TTTCCTCAGATGTTTGATTCT**TCT**AACACATTGTTACCAGCAG 3'
STR1 C305S rev	5' CTGCTGGTAACAATGTGTT**AGA**AGAATCAAACATCTGAGGAAA 3'
STR1 C333S for	5' CCTATTATGGCCTCG**TCT**GGGACTGGTGTAACA 3'
STR1 C333S rev	5' TGTTACACCAGTCCC**AGA**CGAGGCCATAATAGG 3'
STR1 C340S for	5' ACTGGTGTAACA**GCT**AGCATCTTGGCAATGGGG 3'
STR1 C340S rev	5' CCCCATTGCCAAGATGCT**AGC**TGTTACACCAGT 3'
STR2 for2	5' CCCCCCCCCATATGAAGAGAGCATTCAGCTCA 3'
STR2 rev	5' CCCCGGATCCTTATGAAGAAGAACCCACTAT 3'
STR2 C117S for	5' GCTTTTGCTGCTGGT**TCT**TCTGCTCTTGGAATC 3'
STR2 C117S rev	5' GATTCCAAGAGCAGA**AGA**ACCAGCAGCAAAAGC 3'
STR2 C260S for	5' ATACCCGGTAGCAAG**TCT**GTCCCTTTTCCCCTA 3'
STR2 C260S rev	5' TAGGGGAAAAGGGAC**AGA**CTTGCTACCGGGTAT 3'
STR2 C298S for	5' CCGATTGCGGCCTCA**TCT**GGAACCGGTGTAACA 3'
STR2 C298S rev	5' TGTTACACCGGTTCC**AGA**TGAGGCCGCAATCGG 3'
STR2 C305S for	5' ACCGGTGTAACAGCT**TCT**ATTTTGGCATTGGGA 3'
STR2 C305S rev	5' TCCCAATGCCAAAAT**AGA**AGCTGTTACACCGGT 3'

Figure S1

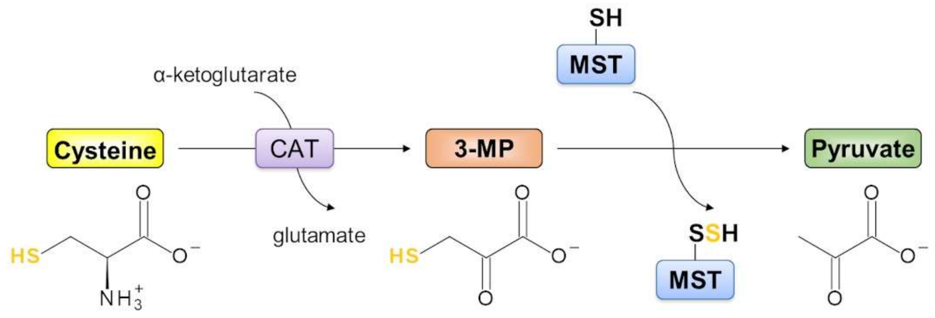

Figure S1. Role of 3-mercaptopyruvate sulfurtransferase (MST) in cysteine degradation.
L-cysteine is transaminated by a cysteine aminotransferase (CAT) (so far unknown in plants) which catalyzes the transfer of the L-Cys amino group to α-ketoglutarate, generating 3-mercaptopyruvate (3-MP) and glutamate as products. 3-MP is then transformed into pyruvate by MST which becomes persulfidated concomitantly on its catalytic cysteine residue.

Figure S2

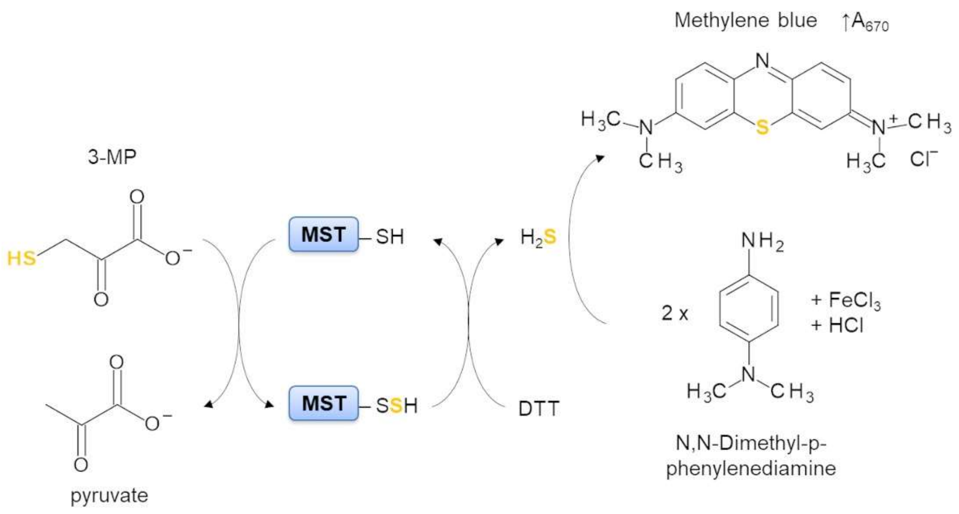

Figure S2. Schematic representation of hydrogen sulfide quantification using the methylene blue assay.

MST catalyzes 3-MP desulfuration to generate an enzyme bound persulfide on the catalytic cysteine and pyruvate. In the presence of a reducing agent like DTT, H_2S is released and reacts in the presence of a mild oxidizing agent (acidified ferric chloride) through an electrophilic aromatic substitution with N, N-dimethyl-p-phenylenediamine directly to methylene blue. Methylene blue displays a characteristic absorbance at 670 nm and therefore allows sulfide quantification (modified after Hartle and Pluth, 2016 (32)).

Figure S3

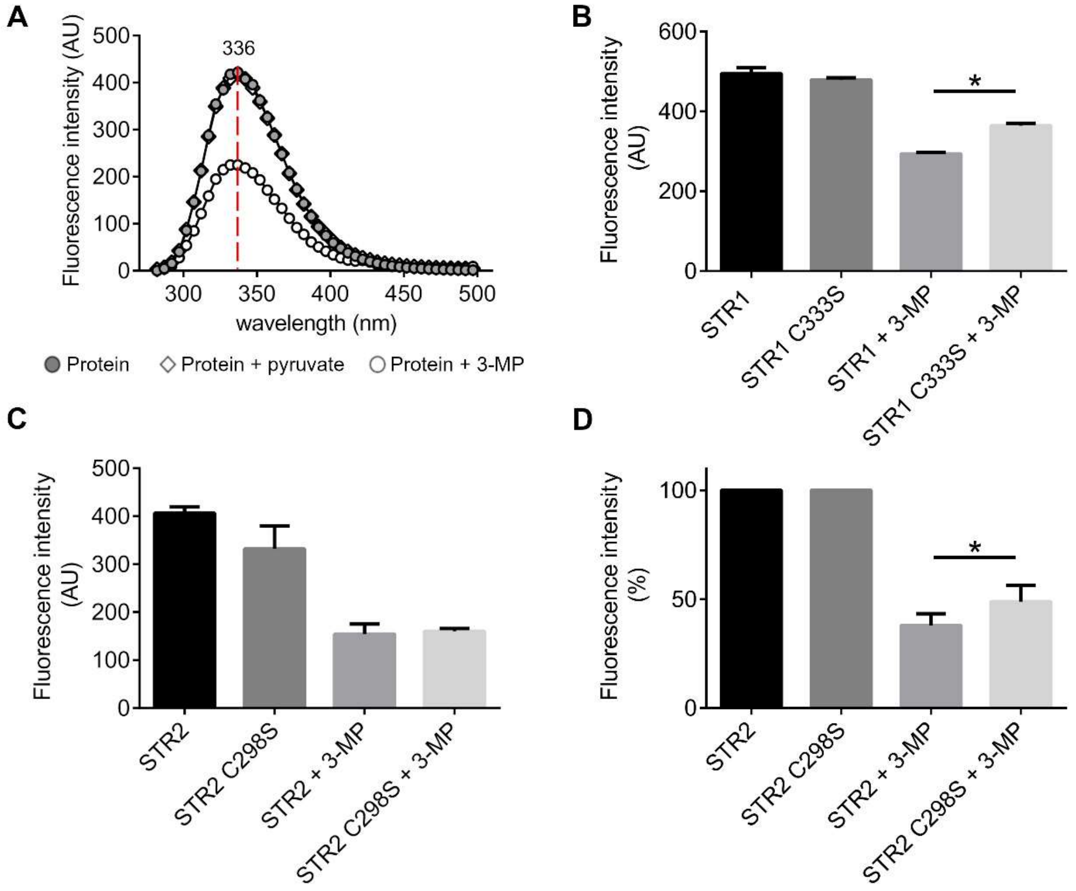

Figure S3. Binding properties of 3-MP to STR1 and STR2.
A: Fluorescence emission spectra of reduced STR1 alone or in presence of a 100-fold excess of 3-MP or pyruvate after excitation at 270 nm. The maximal fluorescence at 336 nm is indicated by a red dashed line.
B-D: The intrinsic fluorescence of 2 µM STR1 (**B**) and STR2 (**C**) and their catalytic variants, alone or with a 100-fold excess of 3-MP, was recorded after excitation at 270 nm. In (**D**), the intrinsic fluorescence of untreated STR2 or the catalytic variant displayed in (**C**) were set to 100% to compare the relative decrease in fluorescence after incubation with 3-MP. For all experiments the mean ± SD (n = 3) is shown**.** The statistical analysis (one-way ANOVA with post hoc Holm-Sidak comparisons for WT vs. catalytic variant) indicated significant changes; *: $P \leq 0.05$.

Figure S4

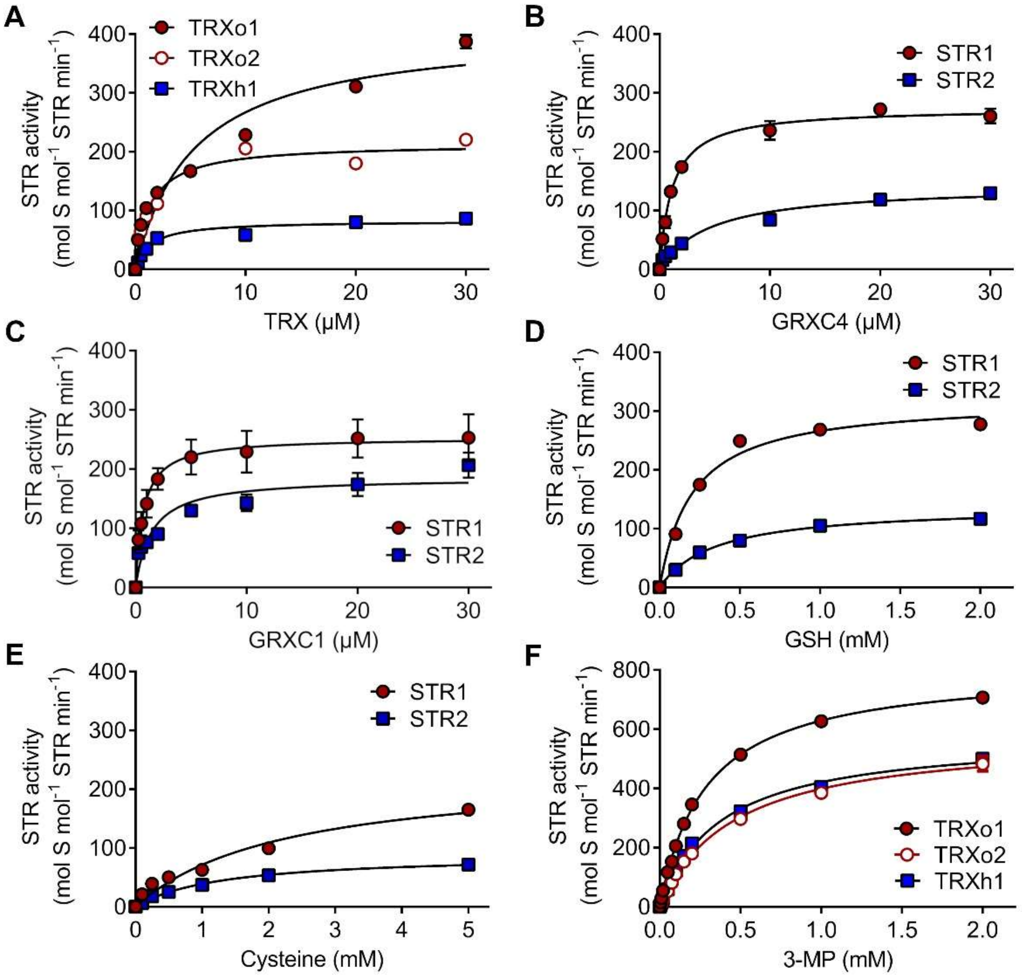

Figure S4. 3-MP sulfurtransferase activity of STR1 and STR2 in the presence of physiological sulfane sulfur acceptors.

Sulfurtransferase activity was measured with 20 nM STR in the presence of 250 µM 3-MP. The reaction mixture contained 0 to 30 µM TRXo1, TRXo2 (for STR1) or TRXh1 (for STR2), 200 µM NADPH and 200 nM NTRB (**A**), 0 to 30 µM GRXC4 or GRXC1, 250 µM NADPH, 250 µM GSH and 0.5 U GR (**B, C**), 0 to 2 mM GSH, 250 µM NADPH and 0.5 U GR (**D**) or 0 to 5 mM cysteine (**E**). The 3-MP concentration dependency of STR activity was measured with 20 nM STR in the presence of 0-2 mM 3-MP as well as 30 µM TRXo1, TRXo2 (for STR1) or 10 µM TRXh1 (for STR2), 200 µM NADPH and 200 nM NTRB (**F**). For all experiments the mean ± SD ($n = 3$) is shown. The activity of STR1 and STR2 is shown in red and blue, respectively.

Figure S5

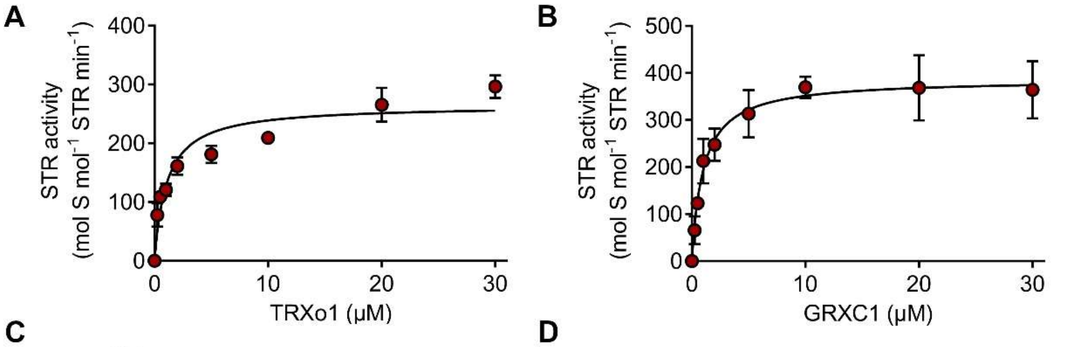

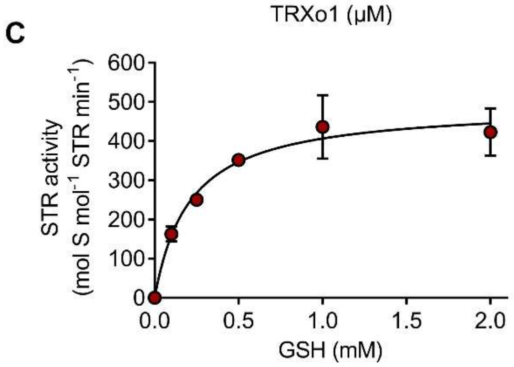

D

	K_m (µM)	k_{cat} (s^{-1})	k_{cat} / K_m (M^{-1} s^{-1})
**TRXo1**	1.1 ± 0.2	4.4	4 x 10^6
**GRXC1**	1 ± 0.16	6.4	6.4 x 10^6
**GSH**	209 ± 43	8.2	3.9 x 10^4

Figure S5. 3-MP sulfurtransferase activity of STR1 in the presence of physiological sulfane sulfur acceptors by following NADPH consumption.
Sulfurtransferase activity was measured with 40 nM STR1 in the presence of 500 µM 3-MP. The reaction mixture contained 0 to 30 µM TRXo1, 250 µM NADPH and 200 nM NTRB (**A**), 0 to 30 µM GRXC1, 250 µM NADPH, 250 µM GSH and 0.5 U GR (**B**), 0 to 2 mM GSH, 250 µM NADPH and 0.5 U GR (**C**). For all experiments the mean ± SD (n = 3) is shown. (**D**) represents the kinetic parameters of 3-MP sulfurtransferase activity of STR1 using distinct sulfur acceptors.

Figure S6

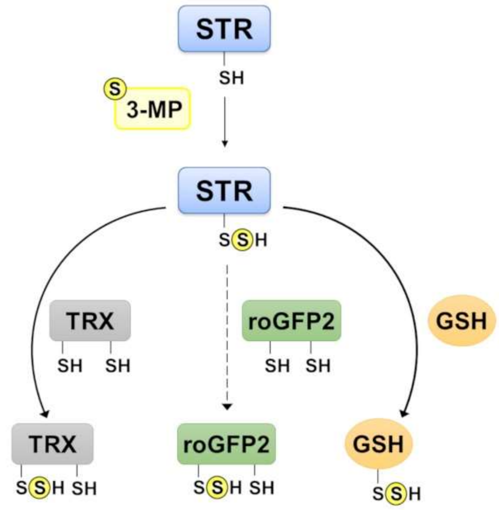

Figure S6. Competition for the STR generated persulfide of different acceptors. The MST is able to transfer the persulfide to roGFP2, here used as a model acceptor protein, but prefers TRX or GSH as acceptors.

Figure S7

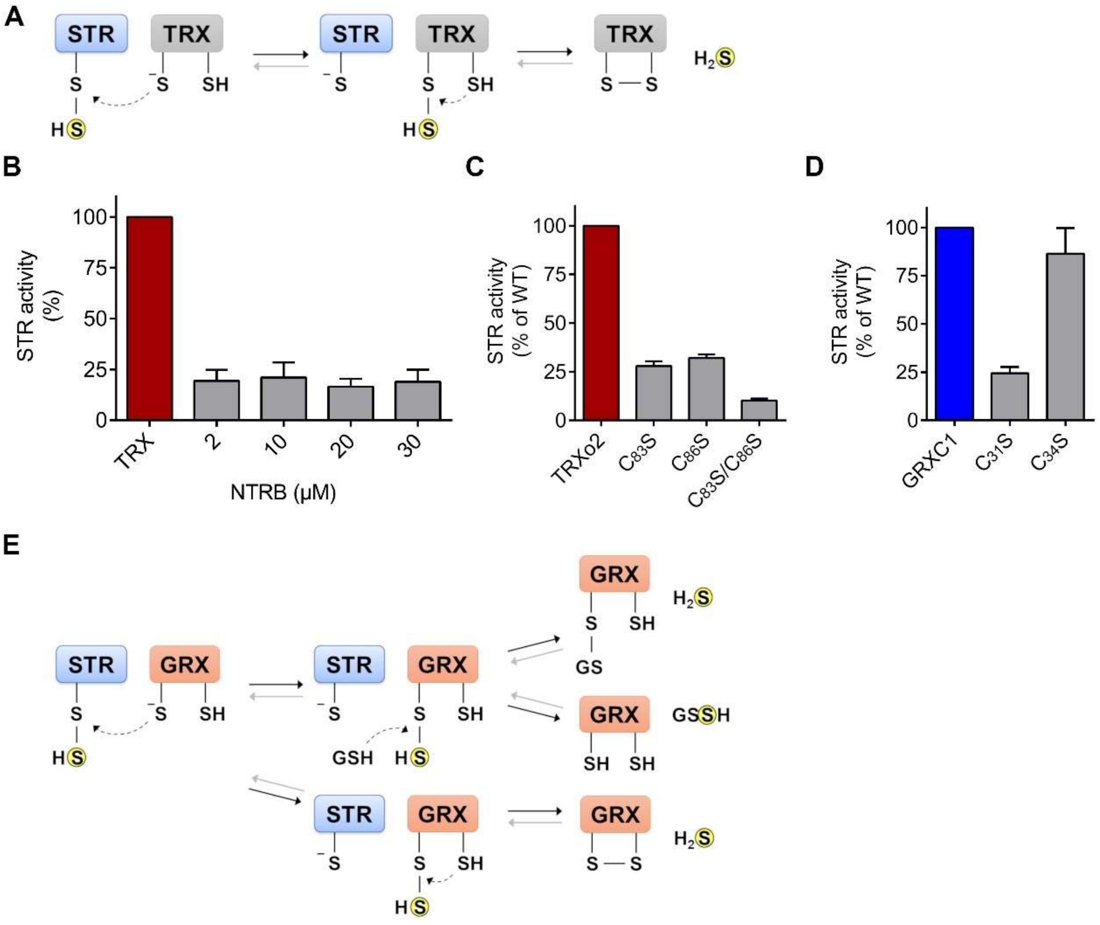

Figure S7. Reduction mechanism of persulfidated MSTs by TRX/GRX systems.

A. Reaction mechanism of MST-mediated H_2S biogenesis through the TRX system. The reaction catalyzed by MSTs comprises two successive sulfur transfer steps. In the first step, the sulfur atom is transferred from 3-MP to the MST, resulting in a cysteine persulfide intermediate in the active site. In the second step, the sulfane sulfur is transferred to TRX leading to the persulfidation of catalytic cysteine before the formation of the intramolecular disulfide bond and the concomitant release of H_2S.

B. Sulfurtransferase activity was measured with 20 nM STR in the presence of 250 µM 3-MP. The reaction mixture contained for the positive control 30 µM TRXo1, 200 µM NADPH and 200 nM NTRB and 200 µM NADPH as well as 2-30 µM NTRB for the other reaction mixtures.

C. The importance of the catalytic and resolving cysteine of TRXo2 for the activity of STR1 was determined in the presence of 250 µM 3-MP, 10 nM STR1 as well as 25 µM TRXo2 and 200 µM NADPH, 200 nM NTRB (n = 3; means ± SD).

D. The importance of the catalytic and resolving cysteine of GRXC1 for the activity of STR2 was determined in the presence of 250 µM 3-MP, 10 nM STR2 as well as 25 µM GRXC1 and 250 µM NADPH, 250 µM GSH and 0.5 U GR. GSH-dependent activity was subtracted to analyze the additional effect of GRX (n = 3; means ± SD).

E. Reaction mechanism of MST-mediated H_2S biogenesis through the GRX system. In the first step, the sulfur atom is transferred from 3-MP to the MST, resulting in a cysteine persulfide intermediate. In the second step, the sulfane sulfur is transferred to GRX. Our experiments do not allow to discriminate what occurs next. First alternative is that the resolving cysteine of GRX is able to reduce the persulfide group on the catalytic cysteine, as TRX does, resulting in the release of H_2S and the formation of an intramolecular disulfide on GRX. Since, a variant for the resolving is as active as the non-mutated GRX in the presence of GSH, a viable alternative is that GSH reacts with the GRX persulfide and either H_2S or GSSH is liberated. Recycling of glutathionylated or disulfide-bridged GRX is not shown here.

Figure S8

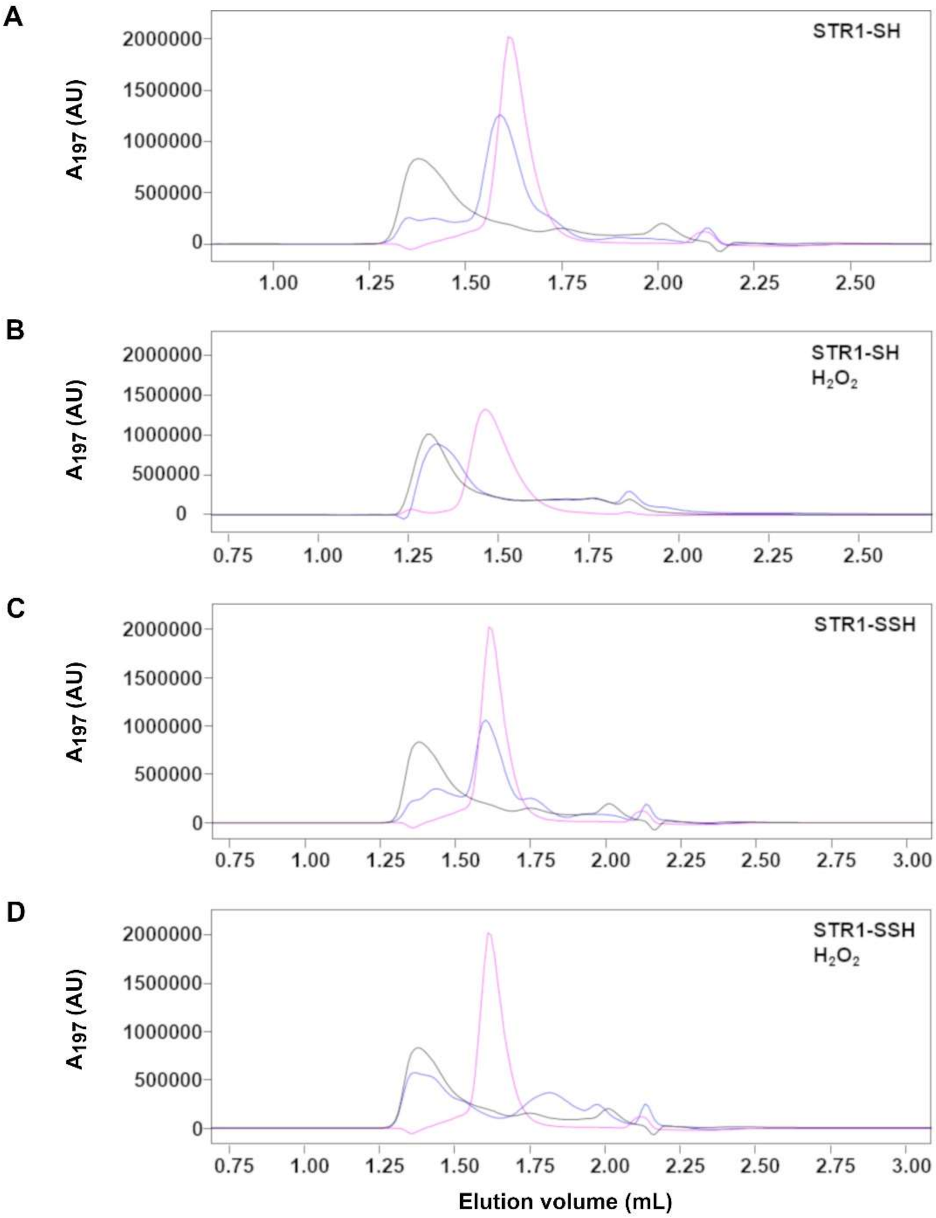

Figure S8. Reactivity of persulfidated STR1.
The influence of STR1 redox state on its capacity to transform 3-MP into pyruvate was analyzed by incubating 500 nM STR1 (reduced (**A**), oxidized with 5 mM H_2O_2 (**B**) persulfidated alone (**C**) and with 5 mM H_2O_2 (**D**)) with 5 mM 3-MP. Product detection was monitored at 197 nm (blue line) after HPLC separation. 3-MP (black line) and pyruvate (pink line) alone were used as controls.
